# The Odorant-Binding Proteins of the Spider Mite *Tetranychus urticae*

**DOI:** 10.3390/ijms22136828

**Published:** 2021-06-25

**Authors:** Jiao Zhu, Giovanni Renzone, Simona Arena, Francesca Romana Dani, Harald Paulsen, Wolfgang Knoll, Christian Cambillau, Andrea Scaloni, Paolo Pelosi

**Affiliations:** 1 Austrian Institute of Technology GmbH, Biosensor Technologies, Konrad-Lorenz Straße, 24, 3430 Tulln, Austria; jiao.zhu@ait.ac.at (J.Z.); Wolfgang.Knoll@ait.ac.at (W.K.); 2 Faculty of Biology, Institute of Molecular Physiology, Johannes Gutenberg-Universität, 55099 Mainz, Germany; paulsen@uni-mainz.de; 3 Proteomics & Mass Spectrometry Laboratory, ISPAAM, National Research Council, 80147 Napoli, Italy; giovanni.renzone@cnr.it (G.R.); simona.arena@cnr.it (S.A.); andrea.scaloni@cnr.it (A.S.); 4 Department of Biology, University of Firenze, Via Madonna del Piano 6, 50019 Sesto Fiorentino, Italy; francescaromana.dani@unifi.it; 5 Department of Physics and Chemistry of Materials, Faculty of Medicine/Dental Medicine, Danube Private University, 3500 Krems, Austria; 6 Architecture et Fonction des Macromolécules Biologiques (UMR 7257), CNRS and Aix-Marseille Université, CDEX 09, 13288 Marseille, France; christian.cambillau@univ-amu.fr

**Keywords:** spider mites, *Tetranychus urticae*, odorant-binding proteins, mass spectrometry, disulfide bridges, ligand-binding

## Abstract

Spider mites are one of the major agricultural pests, feeding on a large variety of plants. As a contribution to understanding chemical communication in these arthropods, we have characterized a recently discovered class of odorant-binding proteins (OBPs) in *Tetranychus urticae*. As in other species of Chelicerata, the four OBPs of *T. urticae* contain six conserved cysteines paired in a pattern (C1–C6, C2–C3, C4–C5) differing from that of insect counterparts (C1–C3, C2–C5, C4–C6). Proteomic analysis uncovered a second family of OBPs, including twelve members that are likely to be unique to *T. urticae*. A three-dimensional model of TurtOBP1, built on the recent X-ray structure of *Varroa destructor* OBP1, shows protein folding different from that of insect OBPs, although with some common features. Ligand-binding experiments indicated some affinity to coniferyl aldehyde, but specific ligands may still need to be found among very large molecules, as suggested by the size of the binding pocket.

## 1. Introduction

Most of the research on agricultural pests is focused on insects, and little work has been performed on Chelicerata. Among these, only a few species such *Ixodes scapularis* and *Varroa destructor* have received more attention, the former because of its impact on human health as carrier of serious diseases [1,2], the latter for the devastating damage caused to honey bees [3,4]. However, spider mites have so far been overlooked, despite the great losses caused by these arthropods in agriculture. In fact, they reproduce very rapidly and feed on a large variety of plants, including wheat, cotton, coffee, orange, lemon, and other fruit trees. Despite their economic importance, the study of chemical communication in mites, but more generally in Chelicerata, has not received much attention, and most reports are limited to behavioral studies. The limited research on Chelicerata chemical communication is perhaps partly justified by the lack of data available for these arthropods at the molecular level. In fact, genome information exists for only a few species, and sequence annotation is scant and incomplete.

Behavioral experiments have shown that chemical communication is very active in these pests, which use volatile compounds to find food, exchange signals between members of the colony, and escape from predators. Scattered information about semiochemicals is available for about twenty species of mites and includes sex, aggregation, and alarm pheromones [5]. In particular, some sex pheromones have been reported. They belong to monoterpenoids or corresponding derivatives, such as α- and β-acaridial [2(*E*)-(4-methyl-3-pentenylidene)-butanedial], the first semiochemicals to be discovered originally in the mites of *Tyrophagus* genus and *Caloglyphus polyphyllae*, and later also in the mite *Rhizoglyphus robini* [6,7,8]. Moreover, 2-hydroxy-6-methylbenzaldehyde was identified as sex pheromone in *Aleuroglyphus ovatus* [9], as well as in other mites and ticks [5]. Finally, citronellol, farnesol, and nerolidol were reported as immature female pheromones in *Tetranychus urticae* [10,11,12], which is the species object of the present work. On the other hand, neral and neryl formate have been identified as alarm pheromones in the mites *Schwiebea elongata*, *Dermatophagoides farinae*, and *D. pteronyssinus* [13,14], while lardolure (1,3,5,7-tetramethyldecyl formate) has been reported as an aggregation pheromone for *Lardoglyphus konoi* and other mites [14,15]. A structurally similar compound [(4R,6R,8R)-4,6,8-trimethyldecan-2-one)] acts as aggregation pheromone for the mite *Chortoglyphus arcuatus* [16]. Apart from pheromones, many natural volatiles produce a repellent reaction on mites. Focusing on *T. urticae*, strong effects have been observed with hydrocarbons such as β-caryophyllene, α-humulene, α-pinene, limonene, and δ-3-carene as well as terpenoids and aromatic compounds such as nerolidol, santalol, carvacrol, cinnamaldehyde, α-terpineol, and thymol [17,18,19,20].

Regarding the proteins used in chemical communication, namely membrane-bound chemoreceptors and soluble binding proteins, Chelicerata in general have only recently been the object of biochemical investigation. Among mites, the honey bee pest *Varroa destructor*, the spider mite *T. urticae*, and the predatory mite *Metaseiulus occidentalis* have had their genomes sequenced [3,21,22]. Odorant receptors (ORs) are not present in the genomes of non-insect arthropods, but ionotropic receptors (IRs) and gustatory receptors (GRs) are well represented. In addition, other receptors, such as those of the degenerin/epithelial Na+ channel family (ENaCs), may be involved in arthropod chemoreception. The genome of *T. urticae* is endowed with only a small number of IRs (19, including a pseudogene), but a very large number of GRs (689 of which 447 active and complete). In addition, 136 ENaCs (108 functioning) seem to compensate for the limited number of IRs [23]. In comparison, the genome of the predatory mite *M. occidentalis* is endowed with numbers of chemoreceptors (65 IRs and 64 GRs) in the same range as those of insects [22].

Three classes of semiochemical carrier proteins are expressed and seem to be active in Chelicerata. The first contains sequences similar to vertebrate Niemann-Pick proteins of type C2 (NPC2). While only one or two genes encode such proteins in vertebrates, which are dedicated to transport of lipids and cholesterol, a wide expansion has been observed in arthropods, with large divergence between sequences [24]. This has been suggested to be caused by environmental pressure to develop a large number of tools for detecting natural odors and pheromones. The few studies so far published on these proteins in Chelicerata and insects seem to support such hypothesis [25,26,27,28]. These proteins exhibit a certain binding capacity for small organic molecules and are expressed in chemosensory organs [25,29].

Recently, genomic and transcriptomic analyses have suggested that lipocalins similar to vertebrate OBPs may also act as carriers for pheromones and odorants in arthropods. This hypothesis is based on the relatively large number of genes expressed in each species as well as on their high expression in antennae and pheromone glands [30]. However, before accepting lipocalins as a second class of soluble binding proteins in arthropods, identification and characterization of these proteins isolated from biological tissues will be necessary.

The third family of putative carriers for semiochemicals in Chelicerata includes polypeptides with a certain (around 15%) sequence identity to insect OBPs and therefore named OBP-like proteins [31]. Such low similarity, however, is still significant when compared to that observed between OBPs of distant orders of insects, such as Lepidoptera and Diptera [32]; accordingly, we shall simply refer to them as OBPs. Chelicerata OBPs present six conserved cysteines, like insect OBPs, but occurring at different sequence positions with respect to the latter proteins. Among Chelicerata, OBPs have been studied in the lone star tick *Amblyomma americanum*, where the first member of this family was discovered [31], and in the mite *Varroa destructor* [33,34,35]. In particular, four of the five transcripts encoding OBPs in *V. destructor* were found to be more abundantly expressed in sensory organs (first pair of legs and mouth parts) than in the second pair of legs, thus suggesting a role for these proteins as semiochemical carriers. The genome of *T. urticae* contains only four sequences encoding OBPs, but at least 47 encoding proteins of the NPC2 family [36]. As a contribution to understanding chemical communication in this species, we have expressed and structurally characterized the four OBPs of *T. urticae* (TurtOBPs), acc. numbers: XP_015786166.1; XP_015794342.1; XP_015782945.1; XP_015791022.1, and performed ligand-binding experiments.

## 2. Results

### 2.1. Protein Sequence Analysis

**Figure 1:**
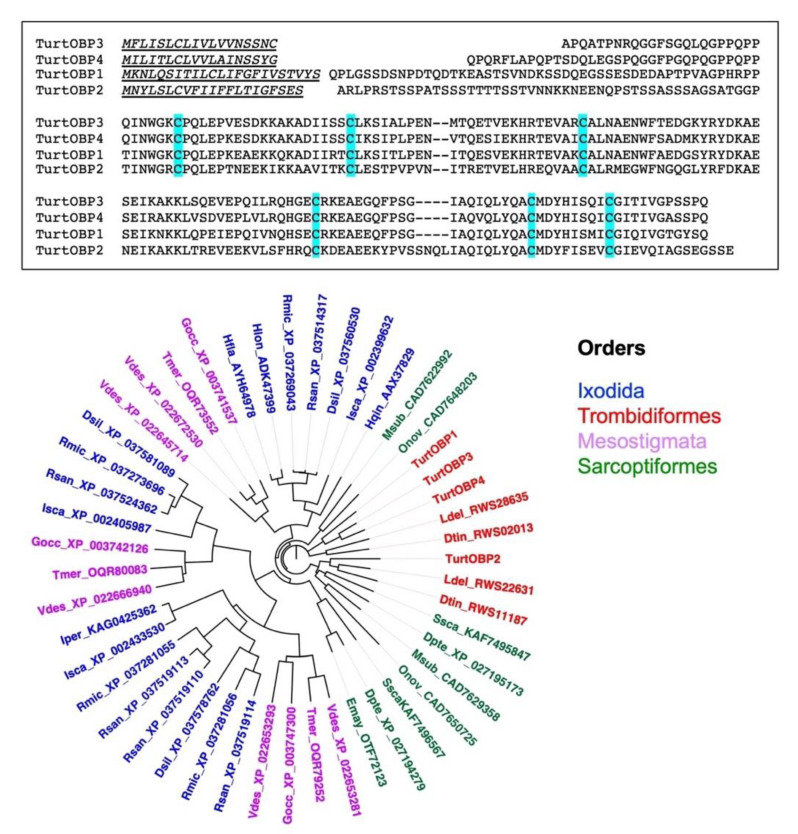
**Upper**: Alignment of the four OBPs of *T. urticae*. Conserved cysteines are highlighted. **Lower**: Phylogenetic tree of the four OBPs of *T. urticae* and their orthologs in ticks and mites. The sequences used to construct the tree are reported in Supplementary Table S1. The tree was built using the neighbor-joining method and Kimura distances on the sequences aligned with ClustalW and default parameters. The tree was visualized with the software FigTree, version 1.4.2 (https://github.com/rambaut/figtree/releases (accessed on 12 March 2021)).

A sample of mixed stages of *T. urticae* was used to extract the total RNA and clone the four OBP sequences reported in the NCBI databases. We confirmed the published sequences, which are aligned in Figure 1. The four TurtOBPs are around 40% identical with each other and contain the conserved motif of six cysteines. 

To put these sequences into a wider frame, we blasted them against the NCBI protein database, limiting the results to “mites&ticks”. As this search produced only two of the five OBPs of *V. destructor*, we then also blasted the other three varroa OBPs and combined the results in the phylogenetic tree reported in Figure 1. All the sequences used in the construction of the tree can be found in Table S1. We can observe that each species is represented by a maximum of five OBP sequences. This could partly be due to incomplete annotation, although such low numbers of genes encoding OBPs have been confirmed in transcriptome studies of *V. destructor* and *Ixodes scapularis* [37]. We also noticed that all TurtOBPs fall within the same cluster, while those of other species, such as *V. destructor* and *I. scapularis*, are distributed among different clusters.

### 2.2. Proteomic Analysis

Next, we checked whether any of the four TurtOBPs encoded in the *T. urticae* genome was expressed at the protein level. Due to the tiny size of these mites, it was impossible to perform any organ dissection. Therefore, we prepared a crude extract of whole mites and separated the proteins by gel electrophoresis under denaturing conditions (SDS-PAGE), as shown in Figure S1. Then, we excised gel bands corresponding to proteins migrating with apparent molecular mass lower than 30 kDa, as most of OBPs and other carrier proteins involved in chemical communication present molecular masses around 20 kDa, and performed corresponding proteomic analysis. Database searching of raw mass spectrometric data allowed identification of 123 proteins (Table S2). Worth mentioning is the fact that in our analysis, we did not find any of the four OBPs encoded in the genome of *T. urticae*, but we identified three (XP_015794572.1, XP_015786143.1, and XP_015788091.1) of the 47 NPC2 proteins predicted by the genome [36]. The absence of TurtOBPs and the small number of NPC2 proteins detected after proteomic analysis might be most likely due to their perhaps being expressed in tiny amounts, below the detection limits of our instrument. These proteins are likely to be expressed only or mainly in chemoreception structures, and the use of whole mites for protein extraction would result in diluting them considerably. It is also possible that in this mite, being highly polyphagous, TurtOBPs and only a small set of NPC2 are expressed at a certain time, depending on the specific environment to be monitored.

On the other hand, proteomic analysis allowed the identification of a protein (XP_015792845) with some similarity to the four TurtOBPs, which presents seven cysteines, three of which can be aligned with those of TurtOBPs. Blasting this sequence against the NCBI database yielded 11 additional members in *T. urticae*, all sharing seven conserved cysteines and about 30–60% identical at the amino acid level. These proteins are well distinct in sequence from the four TurtOBPs, with only 10–15% of identity between members of the two groups. Alignment of the 12 sequences and the four TurtOBPs is reported in Figure S2. Interestingly, this second family of OBPs seems to be specific of *Tetranychus*; in fact, a BLAST search did not return similar entries in any other species of Chelicerata.

Our proteomic analysis also produced four proteins classified as similar to apolipoprotein D (XP_015781506.1, XP_015781509.1, XP_015783215.1, XP_015783283.1) and two fatty acid binding proteins (XP_015782223.1, XP_015783413.1). Recently, we identified relatively large numbers of lipocalins in several species of insect and other arthropods, suggesting that they might represent an additional family of semiochemical carriers [30]. In particular, we reported 49 lipocalin sequences in *T. urticae*, which share between each other variable numbers of residue, from 14 to 76% [30].

### 2.3. Protein Expression and Purification

**Figure 2:**
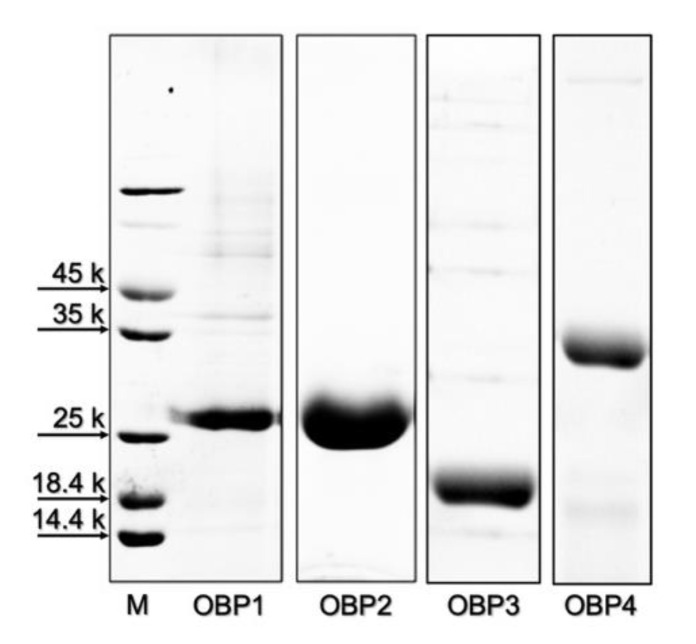
Gel electrophoretic separation under denaturing conditions of the purified samples of the four OBPs of *T. urticae* used for structural (cysteine pairing) and functional (ligand-binding) characterization.

The four TurtOBPs were expressed in bacteria, as reported in the Section 4. After sonication, the proteins were obtained in the supernatant or in the pellet and were purified along with standard procedures by anion-exchange chromatography. Electrophoretic analysis under denaturing conditions (SDS-PAGE) of purified samples is shown in Figure 2.

### 2.4. Cysteine Pairing Assignment

**Figure 3:**
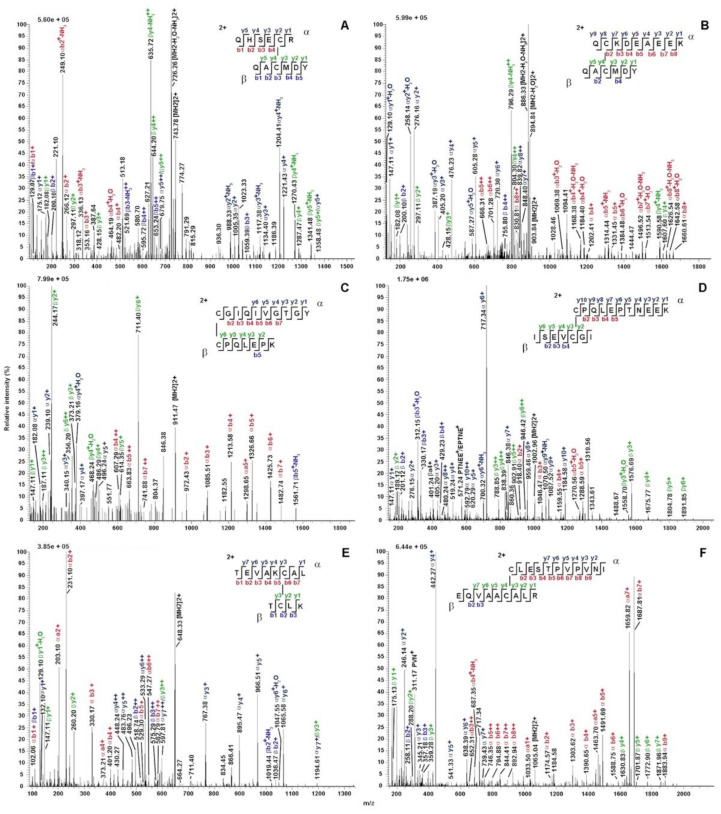
Exemplificative fragmentation spectra of disulfide-bridged peptides identified in the tryptic-chymotryptic digests of TurtOBP1 and TurtOBP2 by nanoLC-ESI-Q-Orbitrap-MS/MS. Panels (**A**,**C**,**E**) and (**B**,**D**,**F**) report peptides from the TurtOBP1 and TurtOBP2 digests, respectively. The fragments are reported in different color depending on peptide present in S-S-linked species and corresponding b and y ion series. Complete data on disulfide-bridged peptides are reported in Table 1.

The four TurtOBPs contain six cysteines that occur at conserved positions (Figure 1), but with a sequence distribution that is different from the typical motif of insect OBPs, suggesting the possibility of a different pairing of possible disulfide bridges [38,39,40]. To ascertain the oxidation state of the cysteines and their eventual pairing, all TurtOBPs were subjected to extensive alkylation with iodoacetamide under non-reducing denaturing conditions. Then, they were subjected to a combination of dedicated proteomic procedures and software analyses that were recently developed for this purpose [41]. Thus, alkylated proteins were initially proteolyzed with trypsin and then subjected to another treatment with chymotrypsin. The resulting peptides were analyzed by nanoLC-ESI-Q-Orbitrap-MS/MS and S-S-crosslinked peptides were identified by dedicated bioinformatics procedures (Table 1). The exhaustiveness of the resulting data was evident for all TurtOBPs. Examples of mass spectra of disulfide-bridged peptides from TurtOBP1 and TurtOBP2 digests are reported in Figure 3. These results and the absence of carboxyamidomethylated peptides in the protein digests demonstrated that all cysteines present in TurtOBPs are involved in disulfide bridges. 

The conserved disulfide pattern identified in TurtOBPs, namely C1-C6, C2-C3, C4-C5 (Table 1 and Figure 3), was different from that of insect OBPs, in which a system of interlocked disulfide bridges (C1-C3, C2-C5, C4-C6) is present. However, it exactly reproduces the arrangement recently observed in the OBPs of the mite *V. destructor* [42]. This phenomenon has two major consequences for the structure of TurtOBPs: (i) the pairing of adjacent cysteines should make *T. urticae* proteins more flexible and less compact than insect OBPs; (ii) linking C1-C6 brings together the protein N- and C-termini, while these two ends generally point in opposite directions in insect OBPs.

### 2.5. Ligand-Binding Experiments

**Figure 4:**
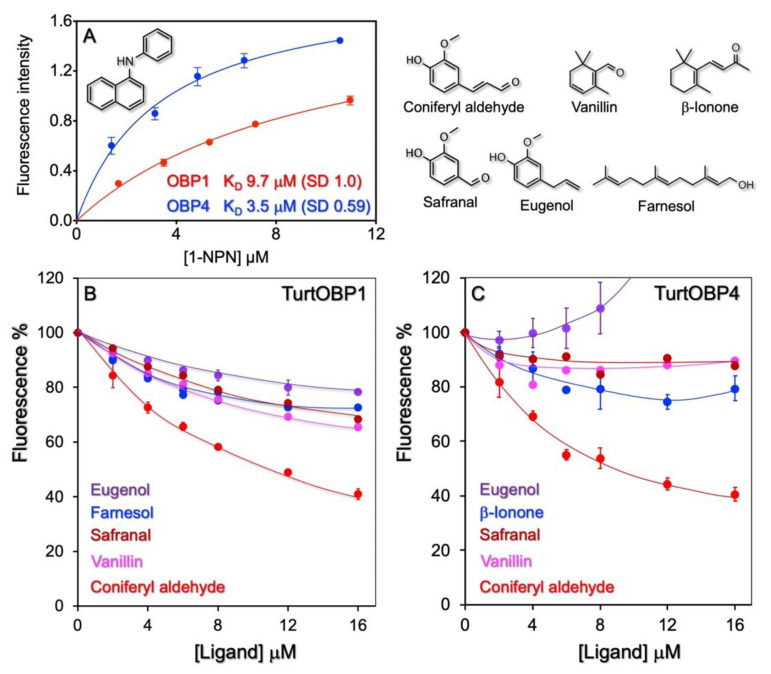
Ligand-binding experiments. (**A**) Only TurtOBP1 and TurtOBP4 bind the fluorescence probe N-phenyl-1-naphthylamine (1-NPN) with moderate dissociation constants, while TurtOBP2 and TurtOBP3 did not produce any change in the spectrum of 1-NPN. (**B**,**C**) Examples of competitive binding experiments performed with TurtOBP1 and TurtOBP4. Tested chemicals are reported in Supplementary Table S3. Only coniferyl aldehyde showed a moderate affinity. For TurtOBP2 and TurtOBP3, we could only monitor quenching of the intrinsic fluorescence of tryptophan (Supplementary Figure S3). The structures of the used ligands are reported in the upper right part of the figure.

Next, we used the purified proteins in ligand-binding experiments to investigate their roles in chemical communication. First, we measured the affinity of the four TurtOBPs to the fluorescent probe 1-NPN. Only for TurtOBP1 and TurtOBP4 did the fluorescence spectrum of 1-NPN undergo the typical blue shift, related to the more hydrophobic environment of the binding pocket and indicating binding of the fluorescent ligand to the protein, which allowed evaluation of the bound probe. For these two proteins, we were able to measure dissociation constants of 9.7 and 3.5 μM, respectively (Figure 4). For TurtOBP2 and TurtOBP3, however, the absence of a blue shift in the spectrum indicated that either the probe did not bind to the protein or that the binding was not accompanied by a measurable change in the spectrum, thus preventing calculation of the affinity constant.

Based on the available information on pheromones, repellents, and other semiochemicals in arthropods, we selected representative terpenoids and other natural compounds (Table S3), which were tested as potential ligands in competitive binding experiments using 1-NPN as the fluorescent reporter. These experiments revealed some moderate ligands among those tested. For example, we measured a moderate affinity of TurtOBP1 and TurtOBP4 to coniferyl aldehyde, with dissociation constants of 9.8 and 6.4 μM. Coniferyl aldehyde is a degradation product of lignin and, therefore, its presence could be a sign that the plant is too old to still represent a source of good food. In any case, the moderate affinity values of the two proteins for this volatile do not allow further speculation. With TurtOBP2 and TurtOBP3, we could only monitor quenching of the tryptophan intrinsic fluorescence caused by the entrance of ligands (Figure S3). In this case, the choice of ligands was limited to aromatic compounds or to chemicals having a system of delocalized π electron, which would be capable of energy transfer with the tryptophan. Altogether, binding experiments only showed limited binding activity of the four OBPs to the tested chemicals. Although our collection of potential ligands covered different chemical classes and compounds with a wide range of size, shape, and polarity (Table S3), we cannot exclude the possibility that good ligand molecules could exist that unfortunately escaped our selection.

### 2.6. Three-Dimensional Folding

To get some insight into potential binding characteristics of TurtOBP1, we built a model using the recently solved structure of *V. destructor* OBP1 (VdesOBP1, PDB ID 7NZA) [42]. Sequence comparison between VdesOBP1 and TurtOBP1 revealed an amino acid identity of 49% over 147 residues without insertions nor deletions (Figure S4A). Furthermore, the disulfide pattern observed in VdesOBP1 is conserved in TurtOBP1. A divergent feature of TurtOBP1 as compared to VdesOBP1 is a N-terminus longer by 35 amino acids. This stretch, however, does not seem to be structured, as reported by the secondary structure prediction server, JPRED4 [43] (Figure S4B). Hence, the structural features observed in VdesOBP1 should be conserved in TurtOBP1.

To verify this hypothesis, we used the automated model building software Swiss-Model homology modeling [44] to generate a TurtOBP1 3D model using the VdesOBP1 X-ray structure P2_1_ form (PDB ID 7NZA) as a matrix. The quality of the model as reported by Swiss-Model was very good, with a Q value of 1.1 and as assessed by Molprobity (Figure S5) [45].

**Figure 5:**
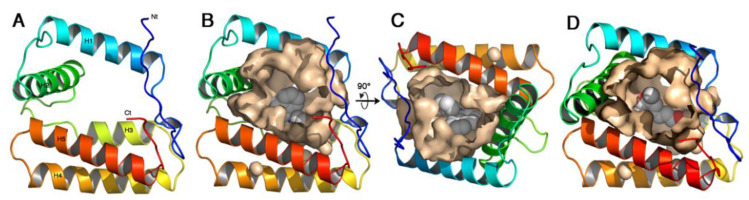
The homology model of TurtOBP1 obtained with Swiss-Model homology modeling [44]. (**A**) Ribbon view of the overall fold with its five α-helices. Colors are in rainbow mode from blue (N-terminus) to red (C-terminus). (**B**) View of TurtOBP1 ribbon with the internal cavity walls and a 1-NPN molecule docked inside. (**C**) Same view 90° from view b. View made with PyMOL (https://pymol.org/ (accessed on 15 March 2021)). (**D**) The homology model of TurtOBP1 showing the internal cavity with coniferyl aldehyde docked inside [45]. View made with PyMOL (https://pymol.org/ (accessed on 16 March 2021)).

As observed in VdesOBP1, the cavity is large and open (Figure 5A). The r.m.s.d. between TurtOBP1 and VdesOBP1 is 0.2 Å, which means that the structures are identical within experimental errors. The cavity volumes of TurtOBP1 and VdesOBP1 are identical, 2156 and 2153 Å^3^, respectively (CASTp calculation). Then, a molecule of 1-NPN was docked into the cavity, with the best position exhibiting a naphthalene stacking interaction on the side-chain of Trp109, and a hydrogen-bond of 1-NPN’s N-H moiety with Glu124 (Figure 5A–C). However, the cavity is much larger than the size of 1-NPN, as observed in the OBP1 of *V. destructor* or in the OBP of the blowfly *Phormia regina* (PDB ID: 5dic, by Ishida, Y., Leal, W.S., Wilson, D.K., unpublished), which exhibits a non-classical OBP fold similar in some way to that of VdesOBP1 [42] and TurtOBP1.

Coniferyl aldehyde was by far the best ligand of TurtOBP1, and we manually docked it within its cavity (Figure 5D). As for NPN, we placed the phenyl ring in a stacking contact with Trp109. The aldehyde moiety is then in hydrogen bond position with Lys128. This position is the most favorable among the many ones that could occur in this large cavity. Superimposition by DALI [46] of TurtOBP1 structure on that of a classical OBP, in this case the OBP/PBP of *Leucophaea maderae* (PDB ID 1ow4 [47]), results in a Z-value of 6.1 and a r.m.s.d. of 3.5 Å. It is worth noting that two disulfide bridges of TurtOBP1 are superimposed with those of LmadPBP: C2-C3 with C1-C3 and C4-C5 with C4-C6. The TurtOBP1 C1-C6 disulfide bridge, instead, is located at the opposite end of the molecule as compared to LmadPBP C2-C5 disulfide bridge (Figure S6).

## 3. Discussion

This study provides an original structural and functional characterization of odorant-binding proteins from an organism belonging to an order in the subphylum of Chelicerata. This is the first time that members of this protein family are subjected to detailed biochemical characterization. The reported results are in perfect agreement with those we recently observed in a parallel research performed on the mite *V. destructor* [42], suggesting the existence of common general features between these proteins in Chelicerata. The main structural differences between Chelicerata and insect OBPs reside in the cysteine pairing and the overall three-dimensional folding of the proteins. From an evolutionary point of view, we can suggest that OBPs of Chelicerata and those of insects may have been generated from a common precursor, although we have not found evidence so far of such ancestor in sequence databases. We were also unable to establish whether it was the different position of the six cysteines in Chelicerata OBPs, with respect to their insect counterparts, to have produced a novel original protein folding or the other way around.

Regarding the binding activity of the four OBPs of *T. urticae*, we could not find a very good ligand for any of them. Moreover, only OBP1 and OBP4 showed that binding of the fluorescent probe 1-NPN was accompanied by a blue shift, as in all the insect OBPs, thus enabling the evaluation of affinities of other ligands in competitive binding assays. In particular, TurtOBP1 and TurtOBP4 showed a moderate affinity to coniferyl aldehyde. Although our selection of putative ligands included common natural compounds, such as pheromones, plant volatiles, and other environmental odors, at present we cannot exclude that chemicals structurally different from those tested here might prove to be good targets for TurtOBPs. Further studies have to be accomplished in this context.

The three-dimensional model of Turt-OBP1, built on the structure of VdesOBP1, with 49% amino acid identity between the two proteins, shows a core very similar to that of VdesOBP1. However, the N-terminus in TurtOBP1 is extended by 35 additional residues as compared to that of the varroa protein. Worth noting is that in the N-terminus of VdesOBP1, 6 residues in form P2_1_ and 11 residues in form P3_2_21 are not visible in the electron density map, probably due to disorder [42]. What could be the structure and function of such a long N-terminus in TurtOBP1? Several hypotheses can be proposed: (i) as in VdesOBP1, it could simply be a loose end in disordered state; (ii) it might wrap around the structured core of the same protein or of another unit, thus potentially stabilizing homodimeric structures in certain conditions; (iii) it could fold back on the core, thus partially closing the cavity, as observed for some insect OBPs [48]. At the present state of our knowledge, none of these hypotheses can be favored over the others.

Besides the four TurtOBPs characterized in this work, the spider mite *T. urticae* is equipped with other families of binding proteins, each represented by a large number of members. In fact, its genome is endowed with 47 genes encoding proteins of the NPC2 family. For these proteins, evidence has been accumulating to support a semiochemical carrier function [24,25,26,27,33,34,35]. The very large expansion of NPC2s in *T. urticae* is likely related to the polyphagous habits of this species and indicates these proteins as likely candidates for binding and transporting the large variety of odorants released by different organisms. Moreover, our proteomic analysis has identified the expression of a protein similar to the four TurtOBPs, which unveiled a new family of putative binding proteins encoded by 12 genes, well distinct from those characterized in the present study, with sequence identities between members of the two families around 10–15%. Interestingly, they seem to be specific of this spider mite, as we could not find any match in any other species of Chelicerata for which sequence information is available. In addition, lipocalins are similarly represented in this species with 49 genes and are also putative carriers for semiochemicals [30].

Combining all these results and observations, we can recognize that TurtOBPs represent only a small number of tools possessed by the spider mite *T. urticae* and, more generally, by Chelicerata to solubilize and transport semiochemicals as compared to the 12 members of the second OBP family, the 47 NPC2s, and the 49 lipocalins discussed here. The function of these proteins still escapes us, but we cannot exclude that they might bind still unknown ligands with narrowly tuned selectivity. We could not detect any of the four TurtOBPs in our proteomic analysis, but this could be probably due to having used the entire mite to prepare the extract, thus diluting proteins specifically expressed in sensory organs, which account for a very small percentage with respect to the total mass of the body. In *V. destructor*, instead, four of the five OBPs encoded in the genome were detected at the protein level, and their expression was higher in the first pair of legs (equipped with chemosensors) compared to the second pair of legs [35]. The production of proteins is an energy consuming process and strongly suggests that the synthesized polypeptides are used by the organism. Until we find whether OBPs are expressed in *T. urticae* and, if so, where they are secreted, we cannot formulate likely hypotheses, including any extrapolation from what is known in *V. destructor*, given the large evolutionary distance between these two species.

## 4. Materials and Methods

### 4.1. Mites

Mites of *T. urticae* were kindly supplied by the Company Bioplanet (Cesena, Italy), and were reared on bean plants.

### 4.2. Chemicals

All chemicals were purchased from Merck, Austria, and were of analytical grade, ex-cept for methanol used to dilute odorants, which was of spectroscopic grade (Uvasol), and acrylamide (Bio-Rad, Vienna, Austria). Oligonucleotides were custom synthesized at Eu-rofins Genomics (Ebersberg, Germany). All enzymes and kits for DNA purification were provided by New England Biolabs (Ipswich, MA, USA).

### 4.3. RNA Extraction, cDNA Synthesis, Cloning, and Sequencing

Total RNA was extracted from whole adult mites using the Trizma reagent (Merck, Austria) along with the enclosed protocol. Single strand cDNA was synthesized using the qScript cDNA SuperMix kit (Quanta bio) following the provided method. PCR was per-formed using specific primers (custom made at Eurofins Genomics, Ebersberg, Germany), which were designed based on the sequences encoding the four OBPs of *T. urticae* that are available in the NCBI database. Crude amplification products were then ligated into pGEM vector (Promega, Walldorf, Neckar, Germany); positive colonies were selected and custom sequenced at Eurofins Genomics (Ebersberg, Germany).

### 4.4. Proteomic Analysis

About 50 mg of whole mites (mixture of different ages) were homogenized in a mortar with 200 μL of 0.1% (*v*/*v*) trifluoroacetic acid in water. The suspension was centrifuged, and the supernatant was separated by denaturing polyacrylamide gel electrophoresis (SDS-PAGE, Figure S1). The whole gel portion including proteins migrating in the mass range 12–30 kDa was excised and subjected to *in-gel* digestion as previously reported [29]; then, the resulting peptide solution was purified following the STAGE procedure [49]. A 1 µL aliquot of the recovered solution was submitted to a nanoLC-ESI-MS/MS analysis on an EASY-nLC 1200 system (Thermo Scientific) equipped with an Acclaim PepMap RSLC C18 column (150 mm × 75 μm ID; 2 μm particle size; 100 Å pore size, Thermo Fisher Scientific, Waltham, MA, USA) and coupled to a LTQ-Orbitrap mass spectrometer (Thermo Fisher, Bremen, Germany). Chromatographic eluents were 99.9/0.1 (*v*/*v*) water/formic acid (solvent A), and 19.95/79.95/0.1 (*v*/*v*/*v*) water/acetonitrile/formic acid (solvent B). The elution program was set as follows: 0 min, 2% solvent B; 5 min, 2% solvent B; 195 min, 40% solvent B; 205 min, 90% solvent B; 207 min, 90% solvent B; 210 min, 90% solvent B; flow rate was set at 300 nL/min. Mass spectra were acquired in positive ion mode, setting the spray voltage at 1.7 kV, the capillary voltage and temperature respectively at 42 V and 175 °C, and the tube lens at 120 V. Data were acquired in data-dependent mode with dynamic exclusion enabled (repeat count 2, repeat duration 15 s, and exclusion duration 40 s). Survey MS scans were recorded in the Orbitrap analyzer in the mass range of *m*/*z* 350–2000 at a 60,000 nominal resolution at *m*/*z* = 400; then, up to seven of the most intense ions in each full MS scan were fragmented (isolation width *m/z* 1, normalized collision energy 35). Mono-charged ions did not trigger MS/MS experiments.

The raw file was analyzed using the MaxQuant software (version 1.6.10.43) [50] searching data against the protein database of *T. urticae* (NCBI) merged with a set of commonly observed contaminants, namely human keratins, bovine serum proteins, and trypsin. In the parameter section, we set trypsin as the proteolytic enzyme, allowing up to two missed cleavages. The minimum required peptide length was seven amino acids. Carbamidomethylation of cysteine and oxidation of methionine were set as variable modifications. As no labeling was performed, multiplicity was set to 1. Mass tolerance was set to ±4.5 ppm for precursors and to ±0.5 Da for MS/MS fragments. Peptide spectrum match (PSM) and protein identifications were filtered using a target–decoy approach at a false discovery rate (FDR) of 1%. Results were reported in the “proteinGroups” output files, containing the full list of identified proteins (Table S1).

### 4.5. Protein Expression and Purification

The genes encoding TurtOBPs 1, 2, and 3 were subcloned in the expression plasmid pET30 (Promega), while TurtOBP4 was subcloned in PET22b (Promega). After transforming DH5a *E. coli* competent cells and plating, colonies containing the insert, as assayed by PCR, were amplified and sequenced. Expression was induced with IPTG in BL-21 (DE3) *E. coli* competent cells and the culture was grown for additional 3 h, at 37 °C. After centrifugation and sonication, the recombinant proteins were found in the supernatant (TurtOBPs 1 and 4) or in the pellet (TurtOBPs 2 and 3). In the latter case, the proteins were solubilized in 8 M urea and 1 mM DTT for 1 h at room temperature, then dialyzed three times against 50 mM Tris-HCl buffer, pH 7.4. Purification of the solubilized proteins was accomplished by anion-exchange chromatography on DE-52 (Whatman) and HiPrep-Q (GE-Healthcare) columns.

### 4.6. Disulfide Assignment

*T. urticae* OBPs (20–60 μg) were separately solved in 0.1 M tetraethylammonium bicarbonate (TEAB), pH 6.5, containing 4 M guanidinium chloride, and subjected to alkylation with iodoacetamide (0.5 M final concentration) for 30 min, in the dark. Reaction mixtures were added with 6 vol of cold acetone, overnight at −20 °C, centrifuged at 16,000× *g* for 20 min at 4 °C, and the supernatants removed; the resulting pellets were finally vacuum-dried. Then, protein samples were dissolved in 0.05 M TEAB, pH 6.5 (2 µg/μL final concentration), and proteolyzed with trypsin (1:10 *w*/*w* enzyme/substrate); the resulting digests were further treated with chymotrypsin (1:8 *w*/*w* enzyme/substrate). Protein digests were desalted with ZipTip*^®^* C18 (Millipore, Burlington, MA, USA) and vacuum-dried. Peptide mixtures were dissolved in 20 μL of aqueous 0.1% (*v*/*v*) formic acid and analyzed using a nanoLC-ESI-Q-Orbitrap-MS/MS platform. The latter consisted of an UltiMate 3000 HPLC RSLC nano-chromatographer (ThermoFisher Scientific) linked to a Q-ExactivePlus mass spectrometer (Thermo Fisher Scientific) through a nano-spray ion source (Thermo Fisher Scientific) [51]. Peptide separation was achieved with the column, flow rate, and solvents described in the previous section. In this case, the elution program was set as follows: 0 min, 3% solvent B; 45 min, 40% solvent B; 50 min, 80% solvent B; 54 min, 80% solvent B; 55 min, 3% solvent B. The mass spectrometer worked in data-dependent mode in positive polarity, executing a full MS1 scan in the range *m*/*z* 345–1350 at a nominal resolution of 70,000, followed by MS/MS scans of the 10 most abundant ions in high energy collisional dissociation (HCD) mode [52]. MS/MS spectra were acquired in a dynamic *m*/*z* range, with a nominal resolution of 17,500, a normalized collision energy of 28%, an automatic gain control target of 50,000, a maximum ion injection time of 110 ms, and an isolation window of 1.2 *m*/*z*. Dynamic exclusion was set to 20 s.

Raw data files were initially analyzed with Proteome Discoverer v. 2.4 package (Thermo Fisher Scientific), including by Mascot v. 2.6.1 (Matrix Science, UK) and ByonicTM v. 2.6 (Protein Metrics, Cupertino, CA, USA) software. Database searching was performed against a customized database containing *T. urticae* OBPs plus common protein contaminants, trypsin and chymotrypsin. Parameters for database searching were those reported in the previous section plus deamidation at Asn/Gln and pyroglutamate formation at Gln. Mass tolerance was set to ±10 ppm for precursors and to ±0.05 Da for MS/MS fragments [53]. Proteolytic enzyme and maximum number of missed cleavages were set to trypsin, chymotrypsin, and 5, respectively. Proteome Discoverer peptide candidates were assigned only when the criteria reported below were satisfied: (i) protein and peptide false discovery rate (FDR) confidence: high; (ii) peptide Mascot score: >30; (iii) peptide spectrum matches (PSMs): unambiguous; (iv) peptide rank (rank of the peptide match): 1; (v) Delta CN (normalized score difference between the selected PSM and the highest-scoring PSM for that spectrum): 0. Byonic peptide candidates were assigned only when the following criteria were satisfied: (i) PEP 2D and PEP 1D: <10 × 10^−5^; (ii) FDR: 0; (iii) q-value 2D and q-value 1D: <10 × 10^−5^. Disulfide bridge assignment was obtained by searching raw data files with BioPharma Finder v. 4.0 (Thermo Fisher Scientific) and pLink v. 2.3.9 [41] software. Both programs were used enabling the specific function of disulfide bridge attribution and applying the parameters described above for Proteome Discoverer and Byonic analyses. Confident disulfide-bridged peptide identification was considered when BioPharma Finder results showed a confidence score of >95 and/or pLink assignments had an E-value of <10^−10^. Candidate fragmentation spectra were always subjected to manual interpretation and verification.

### 4.7. Ligand-Binding Assays

Affinity of ligands to each of the four TurtOBPs was measured in solution using the competitive fluorescence binding assay (TurtOBP1 and TurtOBP4) or measuring the quenching of tryptophan intrinsic fluorescence (TurtOBP2 and TurtOBP3). Spectra were recorded on a PerkinElmer FL 6500 spectrofluorometer in a right-angle configuration at room temperature. Quartz cuvettes with a 1 cm path were used, and slits were set at 5 nm for both excitation and emission. Binding of the fluorescent probe N-phenyl-1-naphthylamine (1-NPN) was monitored by adding to a 2 μM solution of the protein in 50 mM Tris-HCl buffer, pH 7.4, aliquots of a 1 mM methanol solution of 1-NPN to obtain final concentrations in the range 2–16 μM. The excitation wavelength was 337 nm and intensities were recorded in correspondence with the peak maximum, which occurred around 410–415 nm depending on the protein. This protocol was used for OBP1 and OBP4, while with TurtOBP2 and TurtOBP3, the addition of 1-NPN did not produce any blue shift of the emission peak, thus preventing any accurate measurement of the bound probe. The affinities of other ligands to OBP1 and OBP4 were measured in competitive binding assays by titrating a solution of the protein and 1-NPN, both at the concentration of 2 μM with aliquots of 1 mM solutions in methanol of each ligand to final concentration values of 2 to 16 μM. The affinity to 1-NPN was calculated using Prism software. Dissociation constants of competing ligands were evaluated from the corresponding [IC]_50_ values (the concentration of each ligand halving the initial value of fluorescence), using the equation:

K_d_ = [IC]_50_/1 + [1-NPN]/K_1-NPN_

where [1-NPN] is the concentration of free 1-NPN and K_1-NPN_ the dissociation constant of the complex OBP/1-NPN.

For measuring quenching of intrinsic tryptophan fluorescence (OBP2 and OBP3), a 2 μM solution of the protein in 50 mM Tris-HCl buffer, pH 7.4, was titrated with aliquots of 1 mM methanol solutions of ligands to final concentrations of 2 to 15 μM. The tryptophan was excited at 295 nm and emission signals were measured at about 340 nm. This method did not allow evaluation of dissociation constants.

### 4.8. Secondary Structure Analysis

Sequence alignment of OBP1 from *T. urticae* and *V. destructor* was performed with Multalin [54] and EsPrit [55], which allow superimposing sequence data with secondary structure. The secondary structure analysis of TurtOBP1 has been performed with Jpred4 [43] using standard parameters.

### 4.9. Model Building

Considering the high sequence identity (49%) and the conserved disulfide pattern observed between VdesOBP1 and TurtOBP1, we used the automated model building software Swiss-Model homology modeling [44]. The VdesOBP1 X-ray structure P2_1_ form (PDB ID 7NZA) [42] was used as a matrix. The 1-NPN ligand was obtained from the PDB (PDB Id: 3S0B) [56] and docked in the cavity manually with Coot [57], taking into account hydrophobic contacts and hydrogen bonds. The coniferyl aldehyde structure was taken from the PDB entry 6klj. Figures were made with PyMOL software (https://pymol.org/ (accessed on 15–16 March 2021)).

## Figures and Tables

**Table 1:** **Assigned disulfide bridged-peptides identified in the tryptic/chymotryptic digest of *Tetranychus urticae* OBPs**. Reported are the protein under investigation, amino acid sequence, amino acid position within corresponding protein sequence, experimental/theoretical mass value of the observed parent ion, modified cysteine residue, and software used for characterization. ^§^ Cysteine involved in disulfide bridge; * bridged peptide with aspecific cut; ^#^ software used for identification.

**Tetranychus urticae OBP1**								
**Assigned disulfide bridged-peptides**	**Position**	**MH_2_^2+^ exp./theor.**	**MH_3_^3+^ exp./theor.**	**MH_4_^4+^ exp./theor.**	**Cys-Cys**	**MH^+^ exp./theor.**	**BioPharma Finder^#^**	**pLink^#^**
[C^§^PQLEPK]-[HISMIC^§^GIQIVGTGY]	(57-63)-(170-184)		801.7346/ 801.7341		57-175	2403.1882/ 2403.1868		x
[C^§^PQLEPK]-[C^§^GIQIVGTGY]*	(57-63)-(175-184)	911.4472/ 911.4476	607.9676/ 607.9676		57-175	1821.8867/ 1821.8873		x
[TC^§^LK]-[C^§^ALNAENW]	(76-79)-(102-109)	691.3147/ 691.3162	461.2124/ 461.2134		77-102	1381.6216/ 1381.6247	x	
[TC^§^LK]-[C^§^ALN(deam)AENW]	(76-79)-(102-109)	691.8088/ 691.8078	461.5411/ 461.5418		77-102	1382.6097/ 1382.6078		x
[TC^§^LK]-[TEVAKC^§^AL]	(76-79)-(97-104)	648.3378/ 648.3388	432.5611/ 432.5617		77-102	1295.6678/ 1295.6697		x
[QHSEC^§^R]-[QAC^§^MDY]*	(141-146)-(164-169)	743.7794/ 743.7792			145-166	1486.5510/ 1486.5507		x
[IVNQHSEC^§^R]-[QAC^§^MDY]*	(138-146)-(164-169)	906.8789/ 906.8770			145-166	1812.7500/ 1812.7461		x
[IVNQHSEC^§^R]-[QAC^§^M]*	(138-146)-(164-167)	767.8320/ 767.8318			145-166	1534.6561/ 1534.6559		x
**Tetranychus urticae OBP2**								
**Assigned disulfide bridged-peptides**	**Position**	**MH_2_^2+^ exp./theor.**	**MH_3_^3+^ exp./theor.**	**MH_4_^4+^ exp./theor.**	**Cys-Cys**	**MH^+^ exp./theor.**	**BioPharma Finder^#^**	**pLink^#^**
[C^§^PQLEPTNEEK]-[ISEVC^§^GIEV]*	(56-66)-(174-182)	1117.0222/ 1117.0220	745.0174/ 745.0173		56-178	2233.0366/ 2233.0362	x	x
[C^§^PQLEPTNEEK]- [ISEVC^§^GIEVQIAGSEGSSE]	(56-66)-(174-192)		1060.1510/ 1060.1519	795.3652/ 795.3659	56-178	3178.4373/ 3178.4401	x	x
[C^§^PQLEPTNEEK]-[FISEVC^§^GIEV]*	(56-66)-(173-182)	1190.5564/ 1190.5562	794.0402/ 794.0401		56-178	2380.1051/ 2380.1046	x	x
[C^§^PQLEPTNEEK]-[FISEVC^§^GIEVQIAGSEGSSE]	(56-66)-(173-192)		1109.1745/ 1109.1747	832.1328/ 832.1330	56-178	3325.5078/ 3325.5085	x	x
[C^§^PQLEPTNEEK]-[ISEVC^§^GI]*	(56-66)-(174-180)	1002.9666/ 1002.9665	668.9803/ 668.9803		56-178	2004.9254/ 2004.9252		x
[C^§^L]-[EQVAAC^§^ALR]	(76-77)-(96-104)	596.7946/ 596.7948	398.1990/ 398.1991		76-101	1192.5815/ 1192.5818	x	
[C^§^L]-[EQVAAC^§^AL]	(76-77)-(96-103)	518.7439/ 518.7443			76-101	1036.4800/ 1036.4808	x	
[C^§^LESTPVPVNITR]-[EQVAAC^§^ALR]	(76-88)-(96-104)		796.0782/ 796.0791	597.3106/ 597.3112	76-101	2386.2189/ 2386.2216	x	x
[C^§^LESTPVPV]-[C^§^ALR]*	(76-84)-(101-104)	702.3542/ 702.3550			76-101	1403.7005/ 1403.7021		x
[C^§^LESTPVPVNI]-[C^§^ALR]*	(76-86)-(101-104)		544.2818/ 544.2815		76-101	1630.8297/ 1630.8290		x
[C^§^LESTPVPV]-[EQVAAC^§^ALR]*	(76-84)-(96-104)	951.4754/ 951.4768			76-101	1901.9430/ 1901.9459		x
[C^§^LESTPVPVNI]-[EQVAAC^§^ALR]*	(76-86)-(96-104)	1065.0384/ 1065.0403			76-101	2129.0691/ 2129.0728		x
[C^§^LESTPVPV]-[AC^§^ALR] ]*	(76-84)-(100-104)	737.8720/ 737.8735			76-101	1474.7363/ 1474.7392		x
[C^§^LESTPVPV]-[AAC^§^ALR]*	(76-84)-(99-104)	773.3928/ 773.3920			76-101	1545.7779/ 1545.7763		x
[QC^§^KDEAEEK]- [QAC^§^MDY]	(143-151)-(167-172)	903.8514/ 903.8528	602.9036/ 602.9045	452.4296/ 452.4303	144-169	1806.6951/ 1806.6978	x	x
[Q(pGlu)C^§^KDEAEEK]-[QAC^§^MDY]	(143-151)-(167-172)	895.3404/ 895.3396			144-169	1789.6730/ 1789.6713		x
[QC^§^KDEAEEK]-[QAC^§^MDYF]	(143-151)-(167-173)	977.3867/ 977.3870	651.9271/ 651.9273	489.1972/ 489.1974	144-169	1953.7656/ 1953.7662	x	x
[Q(pGlu)C^§^KDEAEEK]-[QAC^§^MDYF]	(143-151)-(167-173)	968.8734/ 968.8738			144-169	1936.7390/ 1936.7397		x
**Tetranychus urticae OBP3**								
**Assigned disulfide bridged-peptides**	**Position**	**MH_2_^2+^ exp./theor.**	**MH_3_^3+^ exp./theor.**	**MH_4_^4+^ exp./theor.**	**Cys-Cys**	**MH^+^ exp./theor.**	**BioPharma Finder^#^**	**pLink^#^**
[C^§^PQLEPVESDK]-[HISQIC^§^GITIV]*	(30-40)-(143-153)	1213.1086/ 1213.1092	890.0750/ 890.0754		30-148	2425.2093/ 2425.2107	x	
[GKC^§^PQLEPVESDKK]-[HISQIC^§^GITIVGPSSPQ]	(30-41)-(143-159)		1097.8973/ 1097.8958		30-148	3291.6762/ 3291.6719	x	
[C^§^PQLEPVESDKKA]-[HISQIC^§^GI]*	(30-42)-(143-150)	1156.0734/ 1156.0749			30-148	2311.1390/ 2311.1420		x
[C^§^PQL]-[HISQIC^§^GITIVGPSSPQ]	(30-33)-(143-159)		732.0355/ 732.0383		30-148	2194.0910/ 2194.0994		x
[C^§^PQL]-[HISQIC^§^GI]*	(30-33)-(143-150)		443.2220/ 443.2217		30-148	1327.6504/ 1327.6496		x
[ADIISSC^§^LK]-[C^§^AL]	(44-52)-(75-77)	626.8176/ 626.8178	418.2143/ 418.2145		50-75	1252.6273/ 1252.6278	x	
[ADIISSC^§^LK]-[C^§^ALNAENW]	(44-52)-(75-82)	933.9396/ 933.9401			50-75	1866.8713/ 1866.8724		x
[ADIISSC^§^LK]-[C^§^ALNAENWFTEDGK]	(44-52)-(75-88)		848.7293/ 848.7300		50-75	2544.1722/ 2544.1744		x
[ADIISSC^§^LK]-[C^§^ALNAENWF]	(44-52)-(75-83)	1007.4736/ 1007.4743			50-75	2013.9395/ 2013.9408		x
[ADIISSC^§^L]-[C^§^AL]	(44-51)-(75-77)	562.7696/ 562.7703			50-75	1124.5314/ 1124.5328	x	
[QHGEC^§^R]-[QAC^§^MDY]	(114-119)-(137-142)	728.7732/ 728.7740			118-139	1456.5387/ 1456.5402	x	x
[Q(pGlu)HGEC^§^R]-[QAC^§^MDY]	(114-119)-(137-142)	720.2600/ 720.2607			118-139	1439.5121/ 1439.5136		x
[Q(pGlu)HGEC^§^R]-[C^§^MDY]*	(114-119)-(139-142)	620.7130/ 620.7128			118-139	1240.4181/ 1240.4179		x
[QHGEC^§^R]-[YQAC^§^MDY]	(114-119)-(136-142)	810.3044/ 810.3056			118-139	1619.6010/ 1619.6035		x
**Tetranychus urticae OBP4**								
**Assigned disulfide bridged-peptides**	**Position**	**MH_2_^2+^ exp./theor.**	**MH_3_^3+^ exp./theor.**	**MH_4_^4+^ exp./theor.**	**Cys-Cys**	**MH^+^ exp./theor.**	**BioPharma Finder^#^**	**pLink^#^**
[C^$^PQLEPK]-[HISQIC^$^GITIV]	(41-47)-(154-164)	998.0242/ 998.0240	665.6854/ 665.6852	499.5160/ 499.5159	41-159	1995.0406/ 1995.0401	x	x
[C^$^PQLEPK]-[HISQIC^$^GITIVGASSPQ]	(41-47)-(154-170)		841.4300/ 841.4299	631.3245/ 631.3244	41-159	2522.2745/ 2522.2740	x	x
[GKC^$^PQLEPK]-[HISQIC^$^GITIVGASSPQ]	(39-47)-(154-170)		903.1358/ 903.1354	677.6038/ 677.6035	41-159	2707.3918/ 2707.3904	x	x
[C^$^PQLEPK]-[HISQIC^$^G]*	(41-47)-(154-160)	784.8812/ 784.8819	523.5901/ 523.5905		41-159	1568.7546/ 1568.7559	x	x
[GKC^$^PQLEPK]-[HISQIC^$^GI]*	(39-47)-(154-161)	933.9817/ 933.9821	622.9904/ 622.9907		41-159	1866.9556/ 1866.9564	x	x
[ADIISSC^$^IK]-[TEVAIC^$^AL]	(55-63)-(81-88)	883.4575/ 883.4576	589.3076/ 589.3077		61-86	1765.9071/ 1765.9074	x	x
[ADIISSC^$^IK]-[AIC^$^AL]	(55-63)-(84-88)	718.8783/ 718.8783	479.5881/ 479.5881		61-86	1436.7488/ 1436.7487	x	x
[ADIISSC^$^IK]-[HRTEVAIC^$^AL]	(55-63)-(79-88)	1030.0382/ 1030.0376		515.5230/ 515.5227	61-86	2059.0686/ 2059.0674	x	x
[AKADIISSC^$^IK]-[HRTEVAIC^$^AL]	(53-63)-(79-88)		753.4045/ 753.4050	565.3053/ 565.3057	61-86	2258.1979/ 2258.1994		x
[AKADIISSC^$^IK]-[TEVAIC^$^AL]	(53-63)-(81-88)		655.6853/ 655.6850		61-86	1965.0404/ 1965.0394		x
[ADIISSC^$^IK]-[TEVAIC^$^A]*	(55-63)-(81-87)	826.9151/ 826.9156			61-86	1652.8223/ 1652.8233	x	x
[ADIISSC^$^IK]-[RTEVAIC^$^AL]*	(55-63)-(80-88)	961.5079/ 961.5081	641.3412/ 641.3414		61-86	1922.0080/ 1922.0084	x	x
[QHGEC^$^R]-[YQAC^$^MDY]	(125-130)-(147-153)	810.3057/ 810.3057			129-150	1619.6036/ 1619.6035	x	x
[Q(pGlu)HGEC^§^R]-[YQAC^§^MDY]	(125-130)-(147-153)	801.7920/ 801.7924			129-150	1602.5761/ 1602.5769		x
[QHGEC^$^R]-[QAC^$^MDY]	(125-130)-(148-153)	728.7728/ 728.7740			129-150	1456.5378/ 1456.5402	x	x
[Q(pGlu)HGEC^§^R]-[QAC^§^MDY]	(125-130)-(148-153)	720.2608/ 720.2607			129-150	1439.5138/ 1439.5136		x
[QHGEC^$^R]-[AC^$^MDY]*	(125-130)-(149-153)	664.7437/ 664.7447			129-150	1328.4796/ 1328.4816	x	x
[Q(pGlu)HGEC^§^R]-[AC^§^MDY]*	(125-130)-(149-153)	656.2314/ 656.2314			129-150	1311.4549/ 1311.4550		x
[QHGEC^$^R]-[QAC^$^MDYHISQ]*	(125-130)-(148-157)	961.3915/ 961.3908			129-150	1921.7751/ 1921.7737		x

## Data Availability

All data supporting results are included in the Supplementary Files.

## Supplementary Materials

The following are available online at https://www.mdpi.com/article/10.3390/ijms22136828/s1.
