# Supplementary material for: The Odorant-Binding Proteins of the Spider Mite Tetranychus urticae
Source: Int J Mol Sci. 2021 Jun 25;22(13):6828. doi: 10.3390/ijms22136828 (PMC8269058; doi:10.3390/ijms22136828)
Supplement: Supplementary file 1 [file ijms-22-06828-s001.zip › Zhu et al. Suppl Files.pdf]

Supplementary Files

The odorant-binding proteins of the spider mite *Tetranychus urticae*

Jiao Zhu[1,2], Giovanni Renzone[3], Simona Arena[3], Francesca Romana Dani[4], Harald Paulsen[2], Wolfgang Knoll[1,5], Christian Cambillau[6], Andrea Scaloni[3], Paolo Pelosi[1]

[1] Austrian Institute of Technology GmbH, Biosensor Technologies, Konrad-Lorenz Straße, 24, 3430 Tulln, Austria
[2] Faculty of Biology, Institute of Molecular Physiology, Johannes Gutenberg-Universität, 55099 Mainz, Germany
[3] Proteomics & Mass Spectrometry Laboratory, ISPAAM, National Research Council, Napoli, Italy
[4] Department of Biology, University of Firenze, via Madonna del Piano 6, 50019 Sesto Fiorentino, Italy
[5] Department of Physics and Chemistry of Materials, Faculty of Medicine/Dental Medicine, Danube Private University, Krems, Austria
[6] Architecture et Fonction des Macromolécules Biologiques (UMR 6098), CNRS, 13288 Marseille Cedex 09, France

Correspondence to: Paolo Pelosi, Austrian Institute of Technology GmbH, Biosensor Technologies, Konrad-Lorenz Straße, 24, 3430 Tulln, Austria. E.mail: paolo.pelosi@ait.ac.at; ppelosi.obp@gmail.com

Supplementary files

Table S1. Amino acid sequences of ticks and mites OBPs used to construct the tree of Figure 1.

>TurtOBP1 _XP_015786166.1 uncharacterized protein LOC107363453 [Tetranychus urticae]
MKNLQSITILCLIFGFIVSTVYSQPLGSSDSNPDTQDTKEASTSVNDKSSDQEGSSESDEDAPTPVAGPHRPPTINWGKC
PQLEPKEAEKKQKADIIRTCLKSITLPENITQESVEKHRTEVAKCALNAENWFAEDGSYRYDKAESEIKNKKLQPEIEPQ
IVNQHSECRKEAEEQFPSGIAQIQLYQACMDYHISMICGIQIVGTGYSQ
>TurtOBP2 XP_015794342.1 uncharacterized protein LOC107370892 [Tetranychus urticae]
MNYLSLCVFIIFFLTIGFSESARLPRSTSSPATSSSTTTTSSTVNNKKNEENQPSTSSASSSAGSATGGPTINWGRCPQL
EPTNEEKIKKAAVITKCLESTPVPVNITRETVELHREQVAACALRMEGWFNGQGLYRFDKAENEIKAKKLTREVEEKVLS
FHRQCKDEAEEKYPVSSNQLIAQIQLYQACMDYFISEVCGIEVQIAGSEGSSE
>TurtOBP3 XP_015782945.1 uncharacterized protein LOC107360741 [Tetranychus urticae]
MFLISLCLIVLVVNSSNCAPQATPNRQGGFSGQLQGPPQPPQINWGKCPQLEPVESDKKAKADIISSCLKSIALPENMTQ
ETVEKHRTEVARCALNAENWFTEDGKYRYDKAESEIKAKKLSQEVEPQILRQHGECRKEAEGQFPSGIAQIQLYQACMDY
HISQICGITIVGPSSPQ
>TurtOBP4 XP_015791022.1 uncharacterized protein LOC107367785 [Tetranychus urticae]
MILITLCLVVLAINSSYGQPQRFLAPQPTSDQLEGSPQGGFPGQPQGPPQPPQINWGKCPQLEPKESDKKAKADIISSCI
KSIPLPENVTQESIEKHRTEVAICALNAENWFSADMKYRYDKAESEIRAKKLVSDVEPLVLRQHGECRKEAEGQFPSGIA
QVQLYQACMDYHISQICGITIVGASSPQ
>Ssca_KAF7495847 .1 hypothetical protein SSS_793 [Sarcoptes scabiei]
MMAIHQLFLTTVASLWSVMLMTAVVALPQPLNTSTNNKPADSISTASSSSIDSNVPLMASASSPSGQNLRSSSQQPMLLT
LEQQQELLDQLAQLQRQTQQLLAAQQSLAAVAAAAAVNGNNNGVSIGTPLDGNSNNDAGSHSPITSGSIQYHRQQNQQQT
HPTTQQLNNPTSNQQSAAIRNDNIDQGSLEQSQSKSNQLNQMINNAQNSQSQSMSGLPQSPQINWGKCPELEPSEQEKLA
KANVITKCLETTPLPANITRESVEQHREQIAGCALRSEGWFTPNGGYDFSKAEKEIRNKHLQADLENQVLNYHGQCRVES
EEKYPTSNSSIIAQIQLYQACMDYFISDVCGIEVNDTDSPQF
>SscaKAF7496567 .1 hypothetical protein SSS_1960 [Sarcoptes scabiei]
MIANMNLYALAIILLLAHLCDAQSTEKLDKSKLKQQQQLPQRPKIDWGKCPQLEPSEKDIKSKSNILQRCLKENPPPNQD
SITPELIIGHQKNVTECALKIENWFDKKGDYKFEKAEKEIKNKKLSSEMQKALVGSHQDCAEVSKTQKKQSKTPMDVVEQ
VQIYQGCMDAHITQHCQIKIGA
>Dpte_XP_027195173 .1 GATA zinc finger domain-containing protein 10-like
[Dermatophagoides pteronyssinus]
MFRSIIFTLLLAMMMFIIVDANIPNHYHHQQHNTSIKLASSMQQPSTQSIPLQASASQQSNTIMNKQQQQQQHSHQQHQQ
PTLLTLEQQQDLLEQLAQLQRQTQQLLAAQQSLAAVAAAAAVNNNGDANSQTGGNIPASGSANIGHLHHHQHQQQQPNHA
QQFGRQQSINQSNTRSSSSSSSSSSNVGHNNKPIQLQNTNGSDGNNNNSGNNNNNGNSQTTAINNGLPQSPQINWGKCPEL
EPSEQEKLAKANVITKCLETTPLPTNITRESVEQHREQIAACALRSEGWFTQQGGYDFSKAEKEIKNKRLQEDLENQVLN
YHSQCRVESEDKYPTSTNSSIIAQIQLYQACMDYFISDVCGIEVNDSDVPQF
>Dpte_XP_027194279 .1 uncharacterized protein LOC113788996 [Dermatophagoides
pteronyssinus]
MKFSTIFAITLATSSMMMMTVMAANKKNKIVAAASEPSTVSVQPADQTPRRPKINWGRCPQLEPTERDIESKSNILQECL
RQNPPPQSESITQEQIIQHQQKVTECALKIENWFDKNGEYKYSKAEKEIKNKNLAKDMEKKLLESHKQCSQIAKDRSQAV
KNMTIVEQVQVYQGCMDAHITQNCQIEISAV
>Ldel_RWS22631 .1 hypothetical protein B4U80_07214, partial [Leptotrombidium deliense]
PPQQGQQFHPQQQGPPQQQQGPGGEQPPYPGSPQIYWGRCPQLEPTMDEKVKKAAVITKCLETTPVPQNITQESVEVHRE
QVAACALQMEGWFNDKGLYKYDKAESEIKAKRLSKEIEERILSYHKQCKEEAEEKFPVSNKALISQIQLYQACMDYFISD
VCGIDVLRR
>Ldel_RWS28635 .1 hypothetical protein B4U80_06376 [Leptotrombidium deliense]
MLKTVCFLLFAIAAVKCQQQPPEAPQPVPQAPPQPPPQPRINWGQCPQLEPKESDKQAKAKVIQGCLQKIPVPTNITQET
IVQHQREVALCALTTEQWFNEQGKYRYEKAENEIKRKGLQPVIQTRIVFHHNKCQTEAKEKQDVIQEVQMYQACMDYYIA
QICGITVTTQ
>Dtin_RWS11187 .1 hypothetical protein B4U79_15176 [Dinothrombium tinctorium]
MFHFLLLFVCFELIHCAPQRNVRLPEVNPSALPQKPSTPQQLQPQLNGQPRNSPQELFQLQPQAPNVRPQDQSAFPSEGP
NENNRQHHNQPQQPPPPLPPIGPPQISWGQCPQLEPTMEEKIKKAAVITKCLELTPVPTNITRESVELHREQVAACALQM
EGWFNEKGTYRFDKAESEIKNKKLQKDIENRVLTYHKQCKEEAEEKFPISSQNLIAQIQLYQACMDYFISEVCGIEVGPS
APAEF
>Dtin_RWS02013 .1 hypothetical protein B4U79_11820 [Dinothrombium tinctorium]
MWKTVAIVIFFCVLFVDCQQPQEPKINWGKCSKIEPTEQDKQEKAKVIQGCLKQVPLPKNVTRESVAQHQREVAVCALNA
ENWFNEEGKYRYEKAERQIKSKKLEKEIETKILFHHNKCKREAQEKTEIIEEVQLYQACMDYYIAQICEITVN
>Msub_CAD7629358 .1 unnamed protein product [Medioppia subpectinata]
MEGVLTSTRLAISAFASNKDQTLLETVFTHLLVICESGYPFVGSFALRSHLSLSNTQSQLLTKMLPKLFATALLAVALLN
GIHCQQMFSQQPPQAQAPSQPQPQSQQPPATQGPQINWGKCPQLEPSEKEKMTKAQVITKCLETTPLPQNITRDTVELHR
EQIAACALQSEGWFTANNAYDFGKAETEIKNKQLTKQIEDQVLGYHKQCKEEAEEKFPNTKNTVIAQIQLYQACMDYFIS
DVCGIEVNEPEAPQFTGAQL
>Msub_CAD7622992 .1 unnamed protein product [Medioppia subpectinata]
MRFLLSLSVFIVCALLSVSAQKPKNATTIATTTQTSNTTADDKPPTINWGKCPQLEPKDSEKRAKQAVLETCLQKHPIPA
NLTQQSIESHQKAIAECALRAEKWFTKAGNYRFSKAETEIRAKKLQKPIESQLLANHKNCESEAKREFPKTAIIDQIQLY

QACVRHVTRSYGFTAFLTLNIHFQMDFHISNVCEIKVENSK

>Onov_CAD7648203 .1 unnamed protein product [Oppiella nova]
MTSLNAISREVYETTNTLFLCLSAAVSRPQRLSLRLPAPTPPATPTPVSDRQMAPKNGDKSKIKTEGLFARIEGHLPGDQ
SDAEFQTPPTLLILYKSRETFAQKTPKMETLLKVSLFLCIVCAFSAFAAPANKKVDKQTATTTGTTPAANATTDKAPTID
WGKCPQLEPKDAEKRAKAAVLETCLARHPIPANLTQETIESHQKAIAECALRTEKWFTAKGNYRYSKAENEIKAKKLPKD
VESALLAHHKNCESEAKREFASKERVIDQIQLYQACMDYHITSACGIQLKDN
>Onov_CAD7650725 .1 unnamed protein product, partial [Oppiella nova]
MSLWLAEHRQERQEFSATRLAIKHKDQTLLATVFTHLLFFDPNQQLPPFFGGQPSGPPQQHFFNPQQFLVQPNQQFNGQP
PQAQPQLAQSQPSQPQLQAQPPAPQGPQINWGKCPQLEPSEKEKSTKAQVITKCLETTPLPPNITRD
>Vdes_XP_022645714 .1 uncharacterized protein LOC111243821 [Varroa destructor]
MKTFIGLVLVGMAMAAPPASGGPEAGKPPTIEWGKCSQLKPSDSERMTKAAVVEKCVQSLPLPEAGKASQAEIEKHREDV
TTCALKAEGWFDDKGVYKFDRARDEIKNKKLASDIETPVLEKHAECQKEAGEKHTSDYIKQVQLYQACMDFNISQICGIK
VVS
>Vdes_XP_022672530 .1 uncharacterized protein LOC111255133 [Varroa destructor]
MKVFVIIALVGMAKAAPQAPASATPAKVPVIEWGKCEQLKPSESERTSKAAVVDKCLQSLPLPDPEKATQQEIDKHRESV
TTCALKAEGWFDDEGVYKFDRARNEIKNKKLDSEVEEAVLLKHDACQKEATEKHDDYINQVQLYQACMDYNISQICGIKV
MV
>Vdes_XP_022653281 .1 uncharacterized protein LOC111247024 [Varroa destructor]
MKTLVVALLASTACLTAADTATGDHQPDSNQFGRWRTCLKSKIPENKQPGMEACINKPGGTDMSKFRRGLGCVLESYGIVNNHRVDL
TKMRSSASLVTSPELKKAFMECPKDDKNSSLDRSIKCVIDHLETSCSAVKAQG
>Vdes_XP_022653293 .1 uncharacterized protein LOC111247029 [Varroa destructor]
MNAAVYIVTAMLACCCSADTASGDHASDTSQFGKWRTCLADKLPAKKREVFDECFIKPGGTDMNKFRRGLNCVLTSDGLL
KNHKVDLKKMSMTAASAGSPELKKAYENCPKDDKNTSEDRSIKCLIDHLEVDCKLASK
>Vdes_XP_022666940 .1 uncharacterized protein LOC111252778 [Varroa destructor]
MHRSFVLQCCGLLFIVGLALAGPLAKDKSERITPKQTVEEFFTSNWGECEPLKNPLVLPEISDAATWERCRVHIPLSPDQ
VEEALPNLSRCMLKVIGWVRANGNMNLTKYMDYLSQAGLDDKTLELTKTAHEKCSQRALSDGSGDYKEQDKIYVECIFRH
FDKTCPPNFQAAVKQGYIVGITQISFVPRYDEVEVESDTDAETEGQAGPSVADATGEAAANDNANGSGGPQ
>Gocc_XP_003747300 .1 uncharacterized protein LOC100900705 [Galendromus occidentalis]
MKIFFAVALTILVCEVSADTASGDHTSDDSKFGKWRSCLASKLPADKQKVHEDCFKKPGGTDMSKFRRGLSCVLDSYGVV
KDHKIDQAKMASTATSITSPELKKAFTECPKDDKNVSEDRTIKCVIDNLETTCKLST
>Gocc_XP_003742126 .1 uncharacterized protein LOC100907366 [Galendromus occidentalis]
MNTFSCVLLGVLVAGACAGPIAKDKSERISPKQTVEEFFTSNWGSCEPLKNPLVLPEISDASTWERCRAHIPISPEQVEE
ALPNLSRCMLKVIGWVRPNGNMNLSKYMDYLSQAGLDGKTLELTKIAHEKCSSRAFSQAANDYKEQDKIYVECIFRHFDK
TCPPNFQAAVKQGYIVGITQISFVPRYDEVDEAGPVEEDAEPQQTQQEDSNSSIKDSDAQ
>Gocc_XP_003741537 .1 uncharacterized protein LOC100907454 [Galendromus occidentalis]
MKVFVLAAFVGLALAAPQAQQGASGGAPKNPIIEWGKCNQLKPSEGERTSKAAVVDKCLQQLPLPDPEKASQAEIDKHRE
SVTTCALKAEGWFDDKGVYKFDRARNEIKNKKLDSEIESQVLVKHDECQKEATEKHTDFINQVQLYQACMDYNISQICGI
KVMV
>Tmer_OQR73552 .1 hypothetical protein BIW11_09656 [Tropilaelaps mercedesae]
MKVFVVMALMGMAMAAPQAPAAPTSAKAPVIEWGKCDQLKPSENERTSKAAVVDKCLQSLPLPDPEKATQQEIDKHRESV
TTCALKAEGWFDDKGVYKFDRARNEIKNKKLDGEIEAAVLVKHDACQKEATEKHEDYINQVQLYQACMDYNISQICGIKV
MV
>Tmer_OQR79252 .1 hypothetical protein BIW11_05867 [Tropilaelaps mercedesae]
MKTLVIALIAGTACLAAADIATGDHQPDTNQFGQWRTCLKSKIPQDKQATMDNCVKKPGGTDMAKFRRGISCVLESYGVV
NGHRVDLRKMGSTASSVRSPELKKAFQECPKDDKNASLDRSIKCVIDHLETSCPLVKAQG
>Tmer_OQR80083 .1 hypothetical protein BIW11_00054, partial [Tropilaelaps mercedesae]
VEQFFTSNWGECEPLKNPLVLPEISDGSTWERCRAHIPLTPEQVEEALPNLSRCMLKVIGWVRPNGNMNLTKYMDYLSQA
GLDEKTLELTKIAHEKCSQRALSDGAGNYKEQDKIYVECIFRHFDKTCPPNFQAAVKQGYIVGITQISFVPRYDDVEMEV
DSDADAEDPNGAYPGPGADDANASSGGSSNNNNKDDSNNNNKADSNGDAQ
>Emay_OTF72123 .1 hypothetical protein BLA29_010316, partial [Euroglyphus maynei]
QLPRAPKINWGRCPQLEPTQRDIEAKSSILQECLRQNPPPQSESITQEQIIQREYKYEKAEKEIKNKNLAKEMEKVLLES
HKQCSQIAKDRKQAVKNLSNVEQVQIYQGCMDAHITQNCQIQISAV
>Hfla_AYH64978 .1 immunogenic protein [Haemaphysalis flava]
MVAFKAALFICALALPVYAQNAPAAEPQRPEINWGKCPQLQPSKEERQQKALVIDTCLEKVPLPDVEHANETVIQQHRED
VTTCALHSEGWFNKNGQYRFDRARTEILNKKLAAEVEPQVLAKHDECKKEAEEKFAHQFVAQVQLYQACMDYHISQICGI
QIQALPQAGAAPPAQG
>Hlon_ADK47399 .1 immunogenic protein [Haemaphysalis longicornis]
MVAFKAALFICALALPVYAQNAPAAEPQRPDINWGKCPQLQPSKEERQQKALAIDTCLEKVPLPDVEHANETVIQQHRED
VTTCALHSEGWFNKNGQYRFDRARTEILNKKLAAEVEPQVLAKHDECKKEAEEKFAHQFVAQVQLYQACMDYHISQICGI
QIQAGAPAGGVPQQPPQQG
>Hqin_AAX37829 .1 Hq05 [Haemaphysalis qinghaiensis]
MVSLKAVLFVCALAFFTCVGSAPAAEPKQPEVNWGKCPQLKPTEEEKQQKAQVVDQCLDKIPIPDLDTANETVILKHRED
VTTCALKTEGWFNKNGHYRFDRAKTEISNKKLPADVQPQVIAKHDECKKEAETKFPKNFIAQVQLYQACMDHHISQLCGV
KIEGDSHGGHSAGGKKASA
>Isca_XP_002399632 .1 uncharacterized protein LOC8038114 [Ixodes scapularis]
MVAFKVSVAVLLCVGSAFAIPAGGKDLDAAAQTQAGVPPRPQINWGKCPQLQPSENERQQKALVIDQCLDKVPLPDVDTA
NDTVIQQHRQDVTTCALKTEGWFNSNGQYRFDRARTEILNKKLAKDVEPQVLSKHDECKKEAEEKFAKNFVAQVQLYQAC
MDYHISQICGIQIAQAAEAHPQGAQQG
>Isca_XP_002433530 .1 uncharacterized protein LOC8051706 [Ixodes scapularis]

MKILLITVVVCVFAATDASRRSTRRQQFTDWRNCMVEQLPSEKKPIYDACYNRSRGTDMHKFREGLKCVLTDYNLLNGTK
IDLAAMTTRASSVSGKLGQAFTDCPKDDHNRNAAKGVKCIIDRLETDCPVPTGSTAH
>Iper_KAG0425362 .1 hypothetical protein HPB47_027463, partial [Ixodes persulcatus]
QQFNDWRACMVEQLPTEKKPIFEACHNRSRGTDMHKFREGLKCVLTDYNLLSGTKIDLAAMTTRASSVSGKLGQAFTDCP
KDDHNRNASKGVKCIIDRLE
>Isca_XP_002405987 .2 uncharacterized protein LOC8033800 [Ixodes scapularis]
MWTSAGKSALVTLGVLLAVGVGLGDAGPVDPKKTDKPEKQTIHEFFSSNWGACEPLKNPLVIPEMSNPTTWVKCQKHLPE
HPQDIEEYLPRLSRCMLKSTGWVRSNGNMNLSKYLQYLSLLGLDDETMNGTREAHKGCIAKAYKEQDYKTQDKVYVECIF
KFFTKSCSANFQGAVKEGYIVGITQLSFMPRMGPLEVVDDVEAEVSEDDAPQDVLPVTNSTSTS
>Rmic_XP_037269043 .1 uncharacterized protein LOC119160877 [Rhipicephalus microplus]
MVAFKAALILCVLVLPAYAQTAGAEPPRPEINWGKCPQLQPSKEERQQKALVIDTCLEKVPLPDVEHANETVIQQHREDV
TTCALHSEGWFNKNGQYRFDRARTEILNKKLAADVEPKVLAKHDECKKEAEEKFAHQFVAQVQLYQACMDYHISQICGIQ
IQGGAGAPAHG
>Rsan_XP_037514317 .1 uncharacterized protein LOC119390702 [Rhipicephalus sanguineus]
MVAFKAALILCVLVLPAYAQTAGAEPPRPEINWGKCPQLQPSKEERQQKALVIDTCLEKVPLPDVEHANETVIQQHREDV
TTCALHSEGWFNKNGQYRFDRARTEILNKKLAADVEPKVLAKHDECKKEAEEKFAHQFVAQVQLYQACMDYHISQICGIQ
IQGAGGAGAPAHG
>Rsan_XP_037519114 .1 uncharacterized protein LOC119396095 [Rhipicephalus sanguineus]
MKTAVVLVLAACLVAVAMSAGGRHSASREDFSRWRECMITKLPQDRIQTYETCRNQSRGTEMRRFRQGLECVLSSYNLVN
RTDVDLSRMQQAAQNVTQQELRAAFQECPQDENNKKVGKAVKCLIDHLETSCPVPAGVNRE
>Rmic_XP_037281056 .1 uncharacterized protein LOC119174308 [Rhipicephalus microplus]
MKTALVLALTACLVAVAMSVGGRISPSRQDFARWRDCMVAKLPEDKVQTYENCRNHSRGTDMRKFRQGLECVLSSYNLVN
RTDVDLSRMQHLAQNITQPEVRAAFQECAQDENNKKVGKAVKCFIDHLETSCPVPDATKRE
>Rsan_XP_037519110 .1 uncharacterized protein LOC119396094 isoform X1 [Rhipicephalus sanguineus]
MKTAVVLVLVACFVAVATSAKDRATSRRQQFDNWRGCMVQKIPKDKAPVYEACHNRSRGTDMHRFRDGLQCVLSSYNIVN
KNDVNLERMAQLAKDITQQDLKGAFQECPKNDRNKSLQRTVKCVIDHLEKTVPCARWRCWSQGVRR
>Rsan_XP_037519113 .1 uncharacterized protein LOC119396094 isoform X2 [Rhipicephalus sanguineus]
MKTAVVLVLVACFVAVATSAKDRATSRRQQFDNWRGCMVQKIPKDKAPVYEACHNRSRGTDMHRFRDGLQCVLSSYNIVN
KNDVNLERMAQLAKDITQQDLKGAFQECPKNDRNKSLQRTVKCVIDHLEKCPVPDGAAGARE
>Rmic_XP_037281055 .1 uncharacterized protein LOC119174307 [Rhipicephalus microplus]
MKTAVVLLLVTCFVALATSAKDRATNHRQQFDDWRSCMVQKIPVDKVPQYDACHGRSRGTDMHRFRDGLQCVLSSYNIVN
KNDVNLDRMAQLARTITQQDLKSAFEECPKNDRNKHVQRAVKCVIDHLERTCPVPDGAAGSRE
>Rsan_XP_037524362 .1 uncharacterized protein LOC119401543 [Rhipicephalus sanguineus]
MRDQLYHVVLLVTFSWLGSAVAGPLEKPEKGDKEGSKQTIQEFFTSDWGACEPLKNPLVIPEMSDPQTWIRCQKHLPEHP
SDIEEYLPRLSRCMLKSTGWVRSNGNMNLSKYLQYLSLLGLDNETMDSTREAHKGCISKAYKETDFKTQDKLYVECIFKH
FTKVCGPNFQGAVKEGYIVGITQLSFMPRMGPLEVVDDVDDDDVVETPPEETAPRAFNETTPIEP
>Rmic_XP_037273696 .1 uncharacterized protein LOC119165718 [Rhipicephalus microplus]
MRDQLHHVVLLVTFAWLGSAVAGPIEKPEKGDKDGGKQTIQEFFTSDWGACEPLKNPLVIPEMSDPQTWIRCQKHLPDHP
SDIEEYLPRLSRCMLKSTGWVRSNGNMNLSKYLQYLSLLGLDNETMNSTREAHKGCISKAYKETDFKTQDKLYVECIFKH
FTKVCGPNFQGAVKEGYIVGITQLSFMPRMGPMEVVDDVDDDDVVETPPEEPAPRPVNETATIEP
>Dsil_XP_037560530 .1 uncharacterized protein LOC119437592 [Dermacentor silvarum]
MVAFKAALILCALVLPAYAQTAGAEPPRPEINWGKCPQLQPSKEERQQKALVIDTCLEKVPLPDVEHANETVIQQHREDV
TTCALHSEGWFNKNGQYRFDRARTEILNKKLAADVEPKVLAKHDECKKEAEEKFAHQFVAQVQLYQACMDYHISQICGIQ
IQGGPQAGAPAHG
>Dsil_XP_037578762 .1 uncharacterized protein LOC119461497 [Dermacentor silvarum]
MKTAVVLVLTACLVAVAMSAGGRASTSREQFSRWRDCMVGKLPAEKVQTYEDCRNHSRGTEMRRFRQGLECVLSSYNLVN
RNDVDLARMRELAQSVTQDRTEGGLPRVPPGRRQQESRKGCEVRH
>Dsil_XP_037581089 .1 uncharacterized protein LOC119464249 [Dermacentor silvarum]
MREQVSHVVLLVTFAWLGSAVAGPIEKPENGDKIGKQTIQEFFTSDWGACEPLKNPLVIPEMSDPQTWIRCQKHLPEHPS
DIEEYLPRLSRCMLKSTGWVRSNGNMNLSKYLQYLSLLGLDNETMDSTREAHKGCISKAYKETDFKTQDKLYVECIFRHF
TKVCGANFQGAVKEGYIVGITQLSFMPRMGPLEVVDDVDGDDVVETPPGEERVPRPINETAPIEP

Table S3. Ligands tested with the four TurtOBPs

Ligand	OBP1	OBP4	OBP2	OBP3
	Competitive binding		Tryptophan quenching	
1-NPN	v	v	v	v
Coniferyl aldehyde	v	v	v	v
Vanillin	v	v	v	v
β-Ionone	v	v	v	v
Safranal	v	v	v	v
Eugenol	v	v	v	v
Farnesol	v			
(*E*)-β-Farnesene	v	v		v
Methyl jasmonate	v			
Linalool	v	v		
Decanal	v			
(*Z*)-3-Hexenol	v			
Hexanal	v			
Dopamine	v	v		
Decanoic acid	v			
Geranyl acetone	v			v
Z11-Hexadecenal		v		
Farnesol		v		
Oleic acid		v		
8-Pentadecanone		v		
Citral		v		v
Benzyl cyanide			v	v
Methoxyeugenol			v	v
4-Methylcathecol			v	v
p-Isopropylphenol			v	v
α-Methoxycinnamaldehyde			v	
α-Pentylcinnamaldehyde			v	v
Carvacrol			v	v
Methoxyvinylphenol			v	
p-tert-Butylbenzaldehyde			v	v
Thymol			v	
Homovanillic alcohol			v	
Cyclamen aldehyde			v	v
Piperonyl alcohol			v	
Octanoic acid				v
Decanoic acid				v
Dodecanoic acid				v
Methyleugenol				v
β-Caryophyllene	v			v
Geraniol	v			v
Santalol	v			v
β-Pinene	v			v

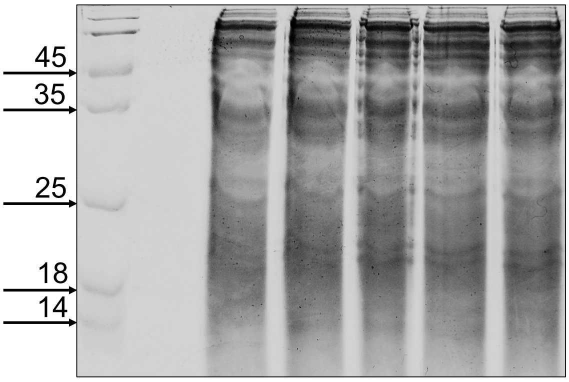

Figure S1. Electrophoretic separation of a crude extract of whole *T. urticae* proteins used for proteomic analysis. The region between apparent molecular mass 12-30 kDa was excised from the five lanes (all loaded with the same sample), pooled and subjected to proteomic analysis as described in the Material and Methods section.

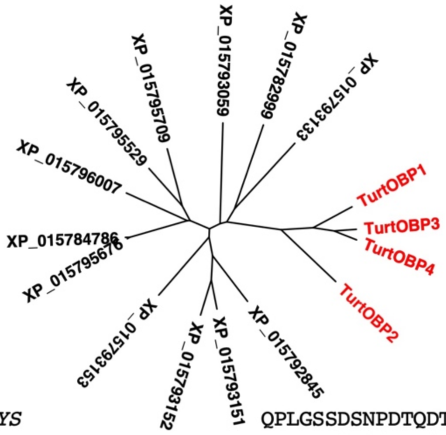


```
XP_015782999 MKILLLMCFIFLTVSSTEA
XP_015793133 MDTFTLSFCFFMLCLSSTLA
XP_015795676 MIKPTFTLILLLFAITLVQS
XP_015784786 MIKPTFTLILLLFALTAVQS
XP_015795529 MIKQTFTLILLIFVISLAQS
XP_015795709 MNQKTFTLFLLIFVISLAQR
XP_015796007 MIKQTFTLILLITTVSLARS
XP_015793151 MIRQSLTLILVLSLISFIHS
XP_015793152 MIRQSLTLILVLSVISSIHS
XP_015792845 MLRQSFTFILLLSVMSFIHS
XP_015793153 MNGQSFILLLIVISSIHC
XP_015793059 MIGQSFTFILLLTVISSTYS
TurtOBP3     MFLISLCLIVLVVNSSNC                                                APQATPNRQG
TurtOBP4     MILITLCLVVLAINSSYG                                     QPQRFLAPQPTSDQLEGSPQG
TurtOBP1     MKNLQSITILCLIFGFIVSTVYS                 QPLGSSDSNPDTQDTKEASTSVNDKSSDQEGSSESDE
TurtOBP2     MNYLSLCVFIIFFLTIGFSES                    ARLPRSTSSPATSSSTTTTSSTVNNKKNEENQPSTS

XP_015782999          GKQTLTNICQELEEFLKNGKFDERRKIRDECALKAFNSEVQES--------LKKCRDILP----------
XP_015793133    DPDGPPHTHTLENLCKDLKESIDDGTFFGRKKILDDCSAKSLPPDQVKA--------MEKCRSMIP----------
XP_015795676       ASDGELVFKEMCNWRTREIKAGTFDEAIKAMNVCREKVLSKDELAA--------VDKCESVVP----------
XP_015784786       ASDGELVFKEICNWRTREIKAGTFDEAIKAMNVCREKVLSKDELAT--------VDKCESVVP----------
XP_015795529       ASDIEQVFKGICDLRAKQIQEGTFDKIIKASSDCREKVLTKDELAA--------IAKCDRILP----------
XP_015795709       APDIRKIFIDFCALRIQRIKEGTFDENNKATNDCREKMLTKDEIAI--------IAKCERILR----------
XP_015796007 ASDNEESTFYVDAFKEVCSLRTKEIKDGNFDKAIKATSDCREKLLSKDELAA--------ISKCEIILP----------
XP_015793151       QPSPAETFNRMCETSLKAIKAGTFEKSIKERQECREKAVPKDVLAA--------AAKCEEAMP----------
XP_015793152       QLSPADVLNQVCETSMKTIKAGTYEKRIKDRQECREKTVPKDVLAA--------AAKCEEAMP----------
XP_015792845       QPNPTEMMNKMCEGGMKAITAGTFEKGIKDRNECREKAVSKEVLAA--------VNKCEELMP----------
XP_015793153    TSIMPDPAKVFNDMCRARLKAIKEGTFEKLIKDREAMKEAGLSKDVLAA--------VNKCEEALP----------
XP_015793059   EKKDMNSDLARFDHICKASLKAIKEGYFDLRINERAECREKAVPENIMTA--------LTKCEATLP----------
TurtOBP3     GFSGQLQGPPQPPQINWGKCPQLEPVESDKKAKADIISSCLKSIALPENMTQETVEKHREVARCALNAENWFTEDGKYR
TurtOBP4     GFPGQPQGPPQPPQINWGKCPQLEPKESDKKAKADIISSCIKSIPLPENVTQESIEKHREVAICALNAENWFSADMKYR
TurtOBP1     DAPTPVAGPHRPPTINWGKCPQLEPKEAEKKQKADIIRTCLKSITLPENITQESVEKHREVAKCALNAENWFAEDGSYR
TurtOBP2     SASSSAGSATGGPTINWGRCPQLEPTNEEKIKKAAVITKCLESTPVPVNITRETVELHRQVAACALRMEGWFNGQGLYR

XP_015782999 --LETSDDVKKVCSDIGTSEPKIEAVKDCVVKNSPP---------EAIEVFKVCLKKSFGRANLM------------
XP_015793133 --LETPSDVEKVCSDIKSHEPKFNEARSCFKSNSPA--------SDAKKTFQDCIKEALDKE---------------
XP_015795676 --MTKADQVTKTCADFNGNKDKYSELVECEKKAVG---------NKIEKFMGCFLAFEKDNEGSSAQKGR-------
XP_015784786 --MTKADQVTKTCADFNGNKDKYSELVECEKKAVG---------NKIEKFMGCFLAFERDNEGSSAQKGR-------
XP_015795529 --LNEADQVIETCGDFNGNKDKFDELIECKEKAVG---------SVGVKYFFCEYDNPFAN----------------
XP_015795709 --INEADQVTEICADFNGIKGKFTELVRCIKQDIG---------STVPKFYFCEYENPFAN----------------
XP_015796007 --MIKADEVTKICNDMNGSLDKFTEQIKCNKQAAG---------DKINKFGECYVAFQRSVAG--------------
XP_015793151 --MVTIDQVNKVCNAKDANLAKFTEVLGCFDKAIGG--------EYADKFSDCCKFMDPENAAKRSK----------
XP_015793152 --MLTADQVNKVCNAKDANLAKFTEVLGCFDKVLGE--------QYTAKFSNCCNLMDPDNDSKRSN----------
XP_015792845 --MSTADQVKQVCSAKDANVAKLTEKLKCEKAALG---------DDMPKFGECCKKINPDNA---------------
XP_015793153 --LMTADQNTKVCGDMKANLPKFTEMVNCIHKAISS--------SNRSALGNDPLFS--------------------
XP_015793059 --MDSQQAVKDACANEAENAPKWAQVWDCKEKAFGK--------NYDAMLAYALCTLNQGAR---------------
TurtOBP3     YTDKAESEIKAKKLSQEVEPQILRQHGECRKEAEGQFPSG----IAQIQLYQACMDYHISQICGITIVGPSSPQ---
TurtOBP4     YTDKAESEIRAKKLVSDVEPLVLRQHGECRKEAEGQFPSG----IAQVQLYQACMDYHISQICGITIVGASSPQ---
TurtOBP1     YTDKAESEIKNKKLQPEIEPQIVNQHSECRKEAEEQFPSG----IAQIQLYQACMDYHISMICGIQIVGTGYSQ---
TurtOBP2     FEDKAENEIKAKKLTREVEEKVLSFHRQCKDEAEEKYPVSSNQLIAQIQLYQACMDYFISEVCGIEVQIAGSEGSSE
```

Figure S2. Alignment of four TurtOBPs with 12 sequences of a putative second OBP family of OBPs in *T. urticae*. Identity values between members of the two groups of proteins are around 10-15% at the amino acid level. Such low similarity is still significant, considering also the alignment of most of the cysteine residues.

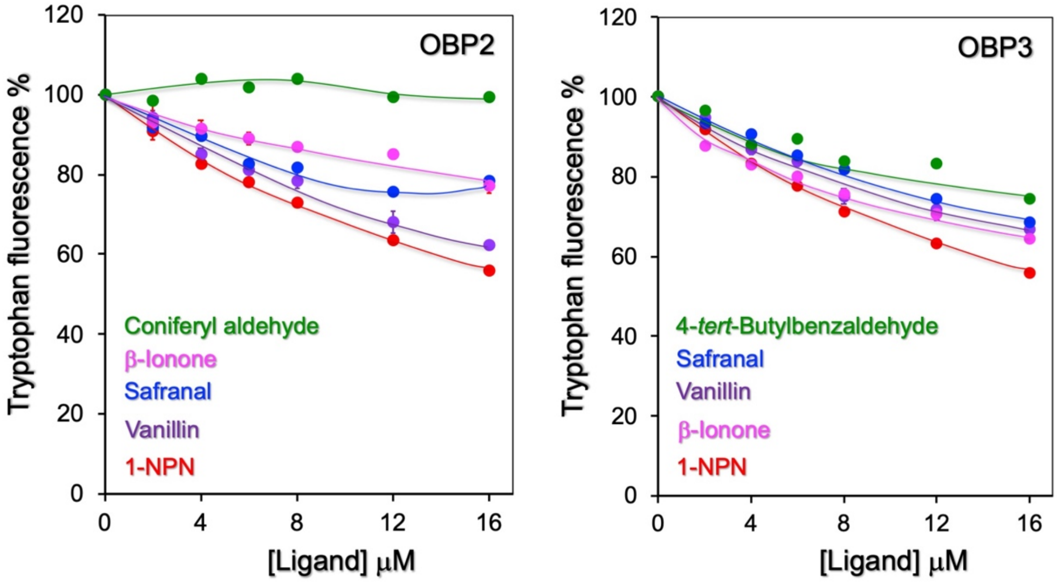

Figure S3. Quenching of the intrinsic tryptophan fluorescence of TurtOBP2 and TurtOBP3 by selected ligands.

A

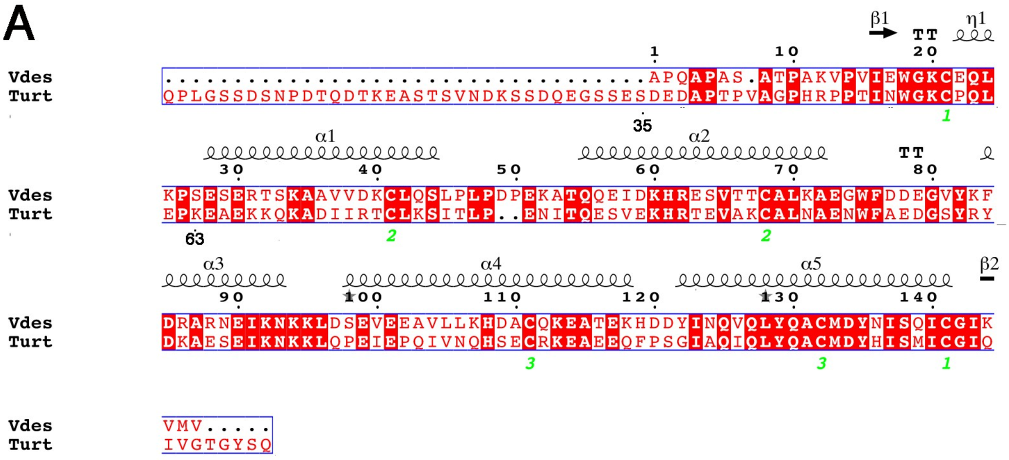

B

```
JPRED Turt

         10        20        30        40        50        60        70        80
---------I---------I---------I---------I---------I---------I---------I---------I
QPLGSSDSNPDTQDTKEASTSVNDKSSDQEGSSESDEDAPTPVAGPHRPPTINWGKCPQLEPKEAEKKQKADIIRTCLKS
~~~~~~~~~~~~~~~~~~~~~~~~~~~~~~~~~~~~~~~~~~~~~~~~~~~~~~~~~~~~~~~HHHHHHHHHHHHHHH~~
                                                                      H1

         90        100       110       120       130       140       150       160
---------I---------I---------I---------I---------I---------I---------I---------I
ITLPENITQESVEKHRTEVAKCALNAENWFAEDGSYRYDKAESEIKNKKLQPEIEPQIVNQHSECRKEAEEQFPSGIAQI
~~~~~~~~HHHHHHHHHHHHHHHHHH~~~~~~~~EEE~~HHHHHHH~~~~~~~HHHHHHHHHHHHHHHH~~~~~HHHHHH
                H2                        H3                  H4

         170       180
---------I---------I------
QLYQACMDYHISMICGIQIVGTGYSQ
HHHHHHHHHHH~~~~~EEEEE~~~~~
 H5
```

Figure S4. Secondary structure analysis of OBP1 from *T. urticae* and *V. destructor.* A. Sequence alignment of these OBPs was perform with Multalin [43] and EsPrit [44], which allow to superimpose sequence data with secondary structure. B. The secondary structure analysis of TurtOBP1 was also performed with Jpred4 [45] using standard parameters.

A

Summary statistics

Protein Geometry	Poor rotamers	1	0.83%	Goal: <0.3%
	Favored rotamers	117	96.69%	Goal: >98%
	Ramachandran outliers	1	0.74%	Goal: <0.05%
	Ramachandran favored	127	93.38%	Goal: >98%
	Rama distribution Z-score	2.53 ± 0.72		Goal: abs(Z score) < 2
	Cβ deviations >0.25Å	0	0.00%	Goal: 0
	Bad bonds:	0 / 1124	0.00%	Goal: 0%
	Bad angles:	7 / 1517	0.46%	Goal: <0.1%
Peptide Omegas	Cis Prolines:	0 / 9	0.00%	Expected: ≤1 per chain, or ≤5%
Low-resolution Criteria	CaBLAM outliers	1	0.7%	Goal: <1.0%
	CA Geometry outliers	0	0.00%	Goal: <0.5%
Additional validations	Chiral volume outliers	0/161		

In the two column results, the left column gives the raw count, right column gives the percentage.
Key to table colors and cutoffs here:

B

MolProbity Ramachandran analysis

model-plm-coot-1.pdb, model 1

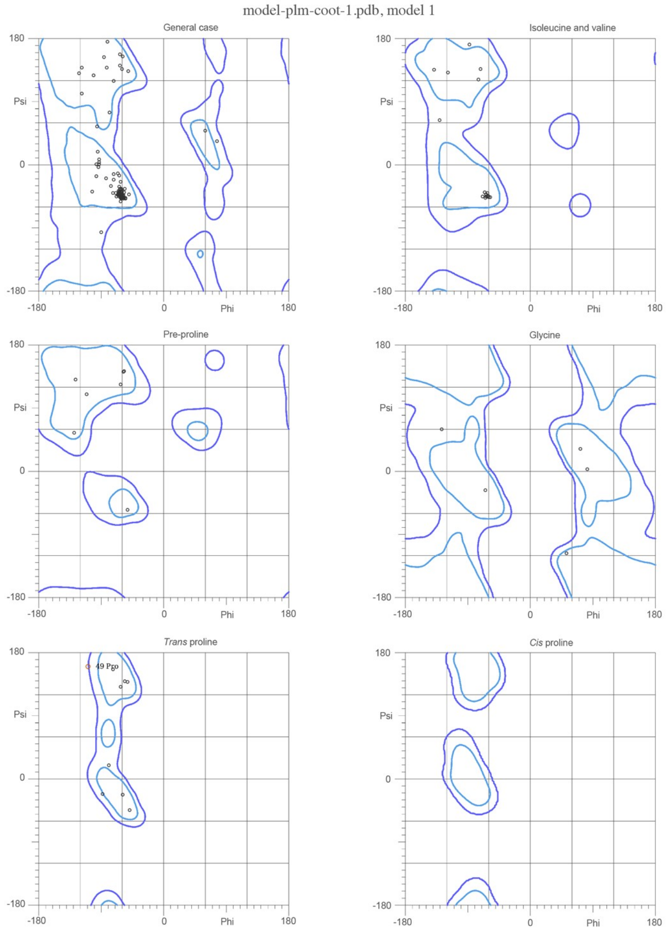

Figure S5. Output files from Molprobity

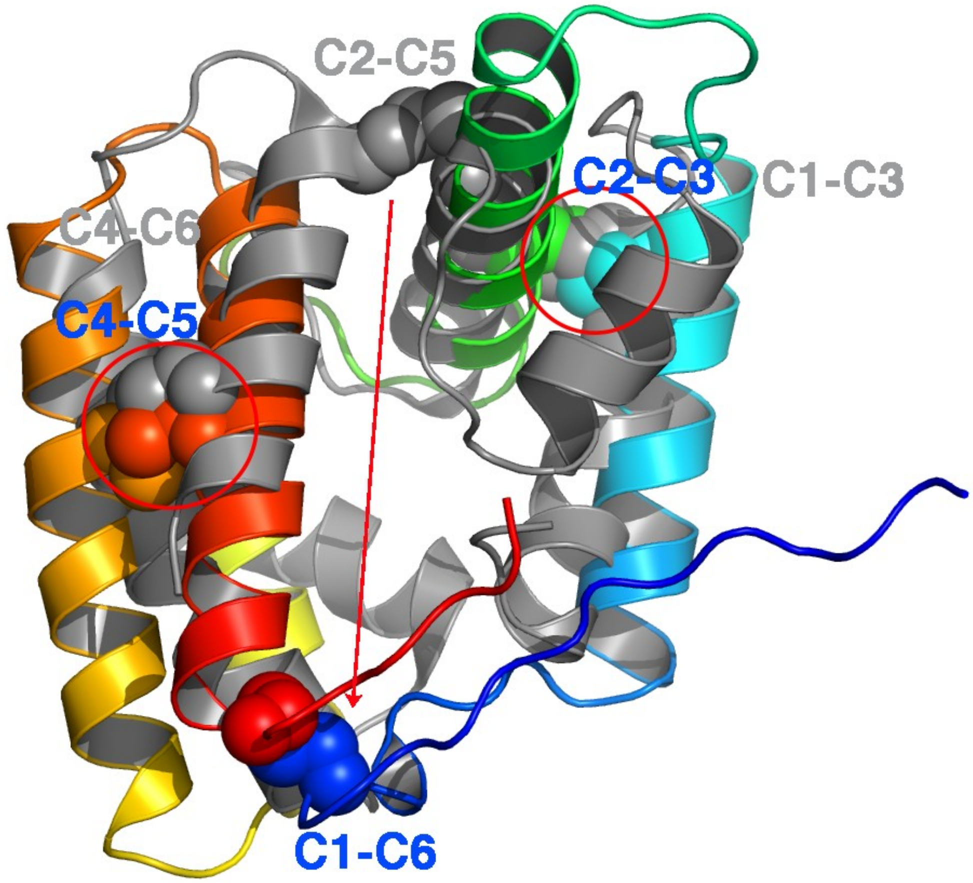

Figure S6. Ribbon view of the structure of TurtOBP1 model superimposed to that of the classical OBP fold of LmadPBP. TurtOBP1 is rainbow colored, LmadPBP is colored grey. The three disulfide bridges of each structure are displayed as atomic spheres. The superimposed disulfide briges are identified by a red circle, while the C2- C5 disulfide of the classical OBP fold is displaced at the opposite end of the molecule in TurtOBP1 (red arrow).
